# Symbolic innovation at the onset of the Upper Paleolithic in Eurasia shown by the personal ornaments from Tolbor-21 (Mongolia)

**DOI:** 10.1038/s41598-023-36140-1

**Published:** 2023-06-12

**Authors:** Solange Rigaud, Evgeny P. Rybin, Arina M. Khatsenovich, Alain Queffelec, Clea H. Paine, Byambaa Gunchinsuren, Sahra Talamo, Daria V. Marchenko, Tsedendorj Bolorbat, Davaakhuu Odsuren, J. Christopher Gillam, Masami Izuho, Alexander Yu. Fedorchenko, Dashdorjgochoo Odgerel, Roman Shelepaev, Jean-Jacques Hublin, Nicolas Zwyns

**Affiliations:** 1 CNRS, Université de Bordeaux, UMR5199 PACEA Bâtiment B2 Allée Geoffroy Saint Hilaire, 33615 Pessac, France; 2 Present Address: Institute of Archaeology and Ethnography, Siberian Branch, Russian Academy of Sciences, 17 Lavrentiev Ave., Novosibirsk, Russia 630090; 3 Archaeology Institute, University of the Highlands and Islands, Kirkwall, UK; 4 Institute of Archaeology, Mongolian Academy of Sciences, Peace Avenue, Ulaanbaatar, 13330 Mongolia; 5 Department of Human Evolution, Max Planck Institute for Evolutionary Anthropology, 04103 Leipzig, Germany; 6 Department of Chemistry “G. Ciamician”, University of Bologna, Via Selmi, 2, 40126 Bologna, Italy; 7 Winthrop University, 701 Oakland Ave, Rock Hill, SC 29733 USA; 8 Faculty of Social Sciences and Humanities, Tokyo Metropolitan University, Hachioji, Tokyo 192-0397 Japan; 9 Institute of Geology, Mongolian Academy of Sciences, Ulaanbaatar, 15160 Mongolia; 10 V.S. Sobolev’s Institute of Geology and Mineralogy, Siberian Branch of Russian Academy of Science, Ak. Koptyug Avenue 3, Novosibirsk, Russia 630090; 11 Chaire de Paléoanthropologie, Collège de France, 75005 Paris, France; 12 Department of Anthropology, University of California-Davis, 1 Shields Avenue, Davis, CA 95616 USA

**Keywords:** Evolution, Archaeology, Cultural evolution

## Abstract

Figurative depictions in art first occur ca. 50,000 years ago in Europe, Africa, and Southeast Asia. Considered by most as an advanced form of symbolic behavior, they are restricted to our species. Here, we report a piece of ornament interpreted as a phallus-like representation. It was found in a 42,000 ca.-year-old Upper Paleolithic archaeological layer at the open-air archaeological site of Tolbor-21, in Mongolia. Mineralogical, microscopic, and rugosimetric analyses points toward the allochthonous origin of the pendant and a complex functional history. Three-dimensional phallic pendants are unknown in the Paleolithic record, and this discovery predates the earliest known sexed anthropomorphic representation. It attests that hunter-gatherer communities used sex anatomical attributes as symbols at a very early stage of their dispersal in the region. The pendant was produced during a period that overlaps with age estimates for early introgression events between *Homo sapiens* and Denisovans, and in a region where such encounters are plausible.

## Introduction

The emergence of the capacity to materialize symbols remains at the center of a persistent debate in the field of Paleoanthropology. The production of abstract drawings and engravings and the use of pigments and personal ornaments are behaviors once thought to be exclusively associated with our species^1–3^, but they could also have emerged gradually out of the multiple cultural and biological interactions between coexisting hominin taxa^4,5^. Early symbolic behaviors, although controversial and episodic, are now reported long before the arrival of modern populations in Eurasia^6–13^, which suggests to some that symbolism emerged following two distinct pathways: one specific to the African Middle Stone Age populations and the other rooted in the Middle Paleolithic and the archaic populations living in Europe and Asia^9,13–17^.

Personal ornaments are a key element of the suite of artefacts usually used as a proxy for early symbolic behaviors^18,19^. Shell beads are attested between 142 and 60 ka in sub-Saharan, East and North Africa and the Levant, but only a few marine species (*Nassarius gibbosulus, Nassarius kraussianus, Glycymeris* sp., *Conus* sp.), among hundreds available along contemporary shores and estuaries, are used as ornaments^20–24^. Although associated human remains have been attributed to anatomically modern humans, they also retain archaic anatomical traits^25^. A clear change in bead-type diversity is observed after 52 ka with the development and proliferation of disk beads, particularly those made from ostrich eggshell in Southern and East Africa during the Later Stone Age (LSA)^26–30^. The picture is drastically different in Eurasia where, from 45 ka cal BP onward, the first uncontroversial personal ornaments show substantial variations in shape, color and raw materials^7,31–33^.

Prior to the Middle to Upper Paleolithic transition in Eurasia, evidence for the production and use of personal ornaments is limited and contested. Some examples include the modification of eagle phalanges, which is believed to have been related to claw extraction^34,35^, the exploitation of large bird feathers^36,37^, and the discovery of a few colored shells and naturally perforated shells with pigment application^13,38,39^ at various sites attributed to Neanderthals between 130 and 50 thousand years ago. While the symbolic significance of these artifacts is well established, there is no clear evidence that they were used as personal ornaments. Personal ornaments made of mammal teeth, mammoth ivory, ostrich eggshell, and tubular bones from small carnivores and birds, as well as different types of soft stone, have been documented in the Upper Paleolithic (UP) of Denisova cave^40,41^, but due to the complex occupation of the site by Neanderthals, Denisovians and *Homo sapiens*, their manufacturer is still the subject of discussion^7^. So far, Bacho Kiro (Bulgaria) is the only Initial Upper Paleolithic (IUP) assemblage for which a clear association between beads and *Homo sapiens* remains is documented^42^. Because these remains have recent Neanderthal ancestors, the variability observed in Eurasian personal ornaments could reflect cultural processes triggered by successive dispersals of modern populations and their encounters with local hominins^7,33,42–44^.

Here we contribute to the debate by reporting the discovery of a pendant found at the Paleolithic site of Tolbor-21, Mongolia. The stratigraphic position, radiometric ages and stone tool assemblage all support an attribution of this object to the Early Upper Paleolithic (EUP) in the broad sense (Supplementary information S1). The application of detailed spectroscopic, microscopic and rugosimetric analyses to the pendant allowed us to document in detail the origin, manufacture and use of the artifact. Although personal ornamentation is well-documented from the Upper Paleolithic of the region^40,45–47^, the pendant is unique in terms of raw material and shape within the regional record. What led to such personal ornamentation diversification in the region is unclear. Given the pivotal age and geographic location of the material considered, the pendant is consistent with an early peopling of Eurasia by Anatomically Modern Humans and an early encounter with other hominins in the region.

### Tolbor-21

**Figure 1:**
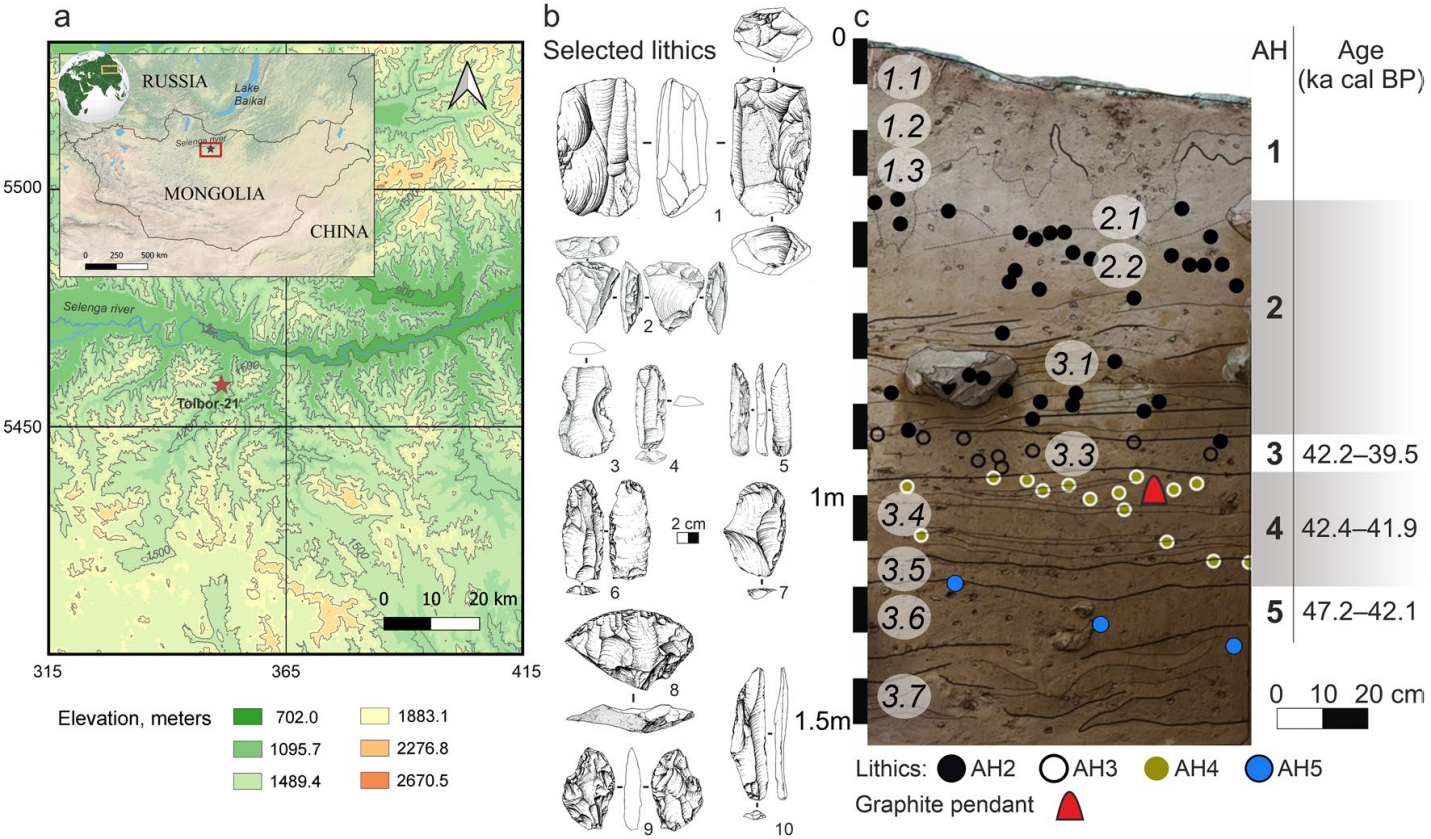
Cultural and chronostratigraphic contexts of the pendant: (**a**) geographic location of the Tolbor-21 site, (**b**) selected artifacts from AH4: 1-subprismatic blade core, 2-convergent Levallois-like flat core, 3, 10-retouched blades, 4, 7-endscrapers, 5-retouched point on blade, 6-truncation, 8-sidescraper, 9-biface. **c,** profile of eastern cross-section of Pit 2 at Tolbor-21 with projected stratigraphic positions of the pendant (red triangle) and lithic artifacts (circles) from archaeological horizons and calibrated radiocarbon dates. Archaeological horizons (AH) are in bold, lithological units (LU) are in italic. The chronological range is established based on radiocarbon dates from Pit 2 using the OxCal 4.4 IntCal20 calibration curve at 68.3% probability interval. Map was created with QGIS 3.8.1. Open Source Geospatial Foundation Project. http://qgis.osgeo.org.

The open-air site of Tolbor-21 (T21) is located in the Northern Khangai Mountains, along the western flank of the Tolbor River valley (1089 m asl), 12 km south of the confluence with the Selenga River (Fig. 1a). Since 2015, our team has conducted excavations in 3 pits and a geological test trench. Pit 2 contains well-stratified Pleistocene deposits with 5 archaeological horizons (AH) attributed to the Upper Paleolithic (UP)^48^, all comprising artifact accumulations of varying density within a matrix of gradually accumulating silt. The lowermost archaeological layers (AH5 through AH3) modelled ages are distributed between 47,230 and 39,530 ka cal BP at 68.3% probability and based on the composition of the archaeological assemblages, they are attributed to the earliest stages of the Upper Paleolithic in Central and Northeast Asia.

AH4, from which the artifact was recovered, is a particularly dense accumulation of lithics. It was identified in all pits with consistent modelled ages ranging between 42,410 and 41,950 ka cal BP at 68.3% probability (for more details on the stratigraphic integrity of the assemblage and its bearing on the age of the pendant , see Supplementary information S2 and Fig. S1, and the method for radiocarbon dating and Bayesian model in Supplementary information S4). In Pit 2, AH4 has yielded 998 piece-plotted lithic artifacts (Supplementary information S3), a fragmentary ostrich eggshell pendant, two ostrich eggshell-beads, three soft-stone pendants, intact and fragmented, and 115 bone fragments (MNI = 6), including *Marmota sibirica*, *Ochotona* sp., *Equus hemionus*, *Equus ferus*, *Bos baicalensis* (yak), and *Coelodonta antiquitatis*^48^.

Overall, the lithic material includes most of the classic traits that define the UP in Eurasia. The technology is oriented toward the systematic production of blades (including small blades/bladelets) which represent 47.6% of the blanks (Fig. 1b n°1, 4–7, 10). Circa 15% of the stone artifacts are retouched; and the tool types include endscrapers (Fig. 1b n°4, 7), sidescrapers (Fig. 1b n°8), various retouched blades and points (Fig. 1b n°5, 6, 10), along with rare bifacial tools (Fig. 1b n°9). The assemblage stands out as a human occupation phase previously unreported in the Valley and in the region. It is technologically and chronologically intermediate between the underlying Initial Upper Paleolithic (IUP) and the Early Upper Paleolithic horizons^49^. Pending further analysis, AH4 is described here as Early Upper Paleolithic (EUP) in the broad sense.

Chronologically the modelled age of layer AH5 ranges from 47,230 to 42,170 cal BP at 68.3% probability (Supplementary information Table S1, Table S2, Fig. S2), coeval with an early *Homo sapiens* presence in the region^44^. The modelled ages for AH3 range between 42,210 and 39,530 cal BP at 68.3% probability, and are in line with dates for the generalization of UP behaviors and the establishment of our species in Eastern Eurasia^50^. The material from AH4 is therefore informative as to regional human dispersals and adaptive responses to MIS3 climatic variation and local environment. The regional synthesis of pendants and personal ornaments presented in this paper attest very early symbolic production in a context of human radiation into novel regions and environments, and introgression with other human species.

## Results

The black pendant with several grooves described here was excavated on the 28th of July 2016 and comes from Pit 2, AH4, square N13 (Fig. 1c). Following initial field identification and piece-plotting as a lithic artifact, the object came to the attention of the team during subsequent lab-based examination, where, after macroscopic observation of the object’s surfaces, it was dry cleaned with a soft brush to remove the remaining sediment dust before analysis. It should be noted that our results preclude trowel damage or other excavation-related modification, and the striations we describe predate burial.

Raman spectroscopy (µ-RS) identifies the black raw material as graphite, formed by high-grade metamorphism of amphibolite or hornblende hornfels facies heated at *circa* 550–560 °C (see Supplementary information S5, Fig. S3). The pendant is 43.4 mm long, 21.4 mm wide, and 13.9 mm thick, with a plano-convex cross-section, a flat side, and a convex side. The convex side is partially broken and covered with white concretions on both the broken part and the intact surface. µ-RS identifies the white concretion as calcite.

**Figure 2:**
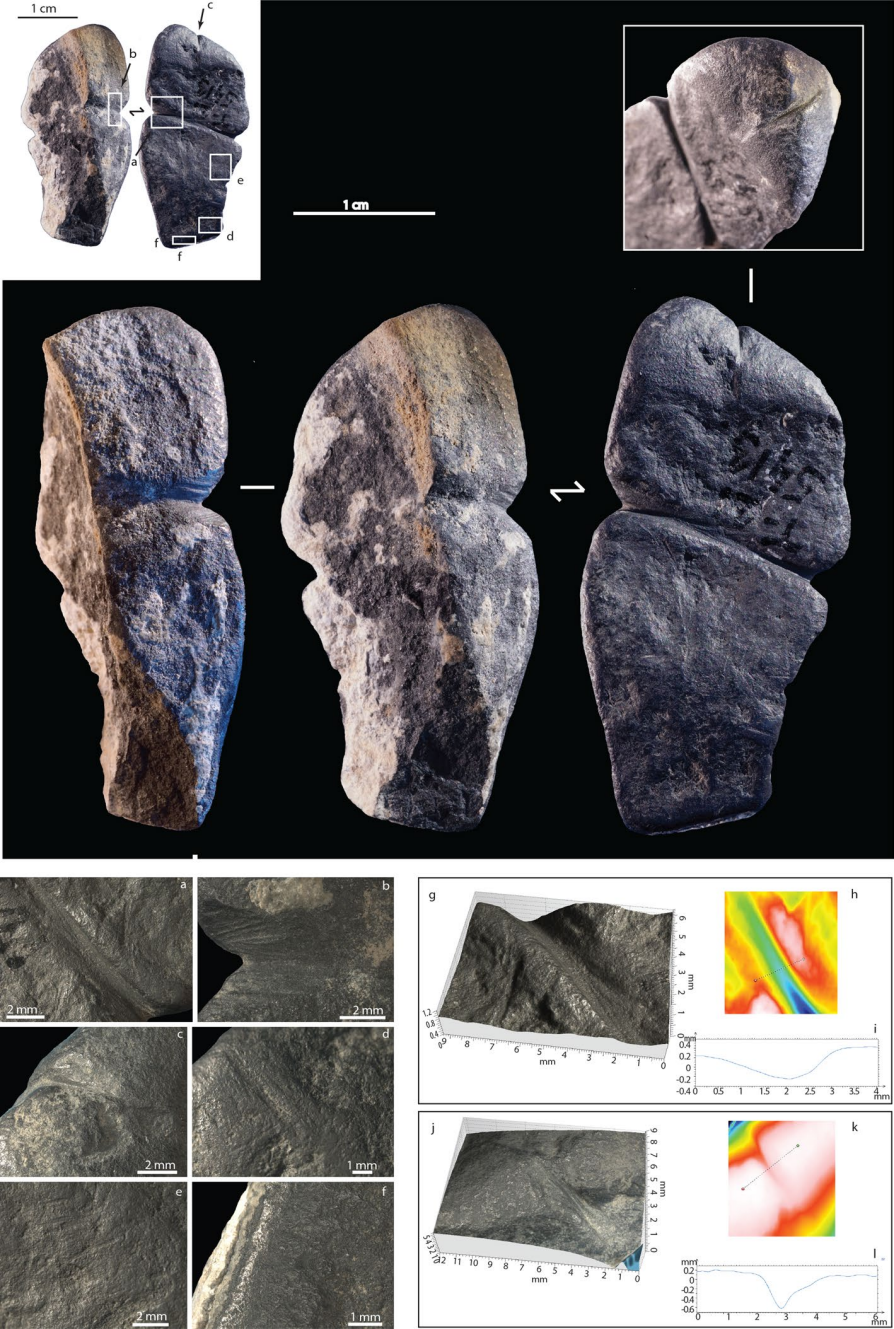
Microscopic images of the modifications observed on the graphite pendant: (**a**–**b**) mid-section groove, (**c**) short groove located at one extremity of the artefact, (**d**–**e**) parallel striations observed on the flat side of the artefact, (**f**) highly smoothed and shiny facet present on the flat side of the artefact, (**g**) 3D reconstruction of the mid-section groove, (**h**–**i**) profile of the mid-section groove, (**j**) 3D reconstruction of the groove located at one extremity of the artefact, (**k**–**l**) profile of the groove located at one extremity of the artefact.

A deep transversal groove crosscuts the flat and the convex sides of the pendant at the mid-section (Fig. 2a). Microscopic analyses show that the groove is covered by thin identical longitudinal and parallel striations. This technological pattern is consistent with repeated scraping by a sharp and pointed object, like a lithic point (Fig. 2a,b). 3D reconstruction of the groove profile is 0.6 mm deep, 2 mm width, with a broad curved section and oblique edges (Fig. 2g). Use-wear is characterized by the smoothed and rounded edges of the striations covering the groove; but also by the smoothed ridges of the groove itself (Fig. 2h,i).

Another short groove of 0.8 mm depth and 0.8 mm width is observed at the extremity of the pendant (Fig. 2c). It is broad with oblique edges and it was made by the repeated scraping of a sharp tool. The edges of the groove are steep (Fig. 2j), while the ridges are sharp (Fig. 2k,l). The smooth, rounded ridges of the mid-section groove contrast with the more sharp-edged appearance of the deep groove at the extremity of the pendant. The flat side of the pendant is covered by short parallel striations indicating that the surface was modified by grinding (Fig. 2d,e). A short shallower groove (0.5 mm) flat at the bottom is visible on the flat side of the pendant (Fig. 2d). The latter appears highly eroded, smoothed and the ridges are rounded. A shiny smoothed facet is also visible on the flat side (Fig. 2f).

A non-invasive tribological analysis was conducted on each archaeological surface of the artifact, including the flat and convex sides and the breakage. The application of multiple standard parameters (ISO 25,178) revealed different textures on each analyzed area, characterized by distinct roughness values (Supplementary information Table S3, Fig. S4).

**Figure 3:**
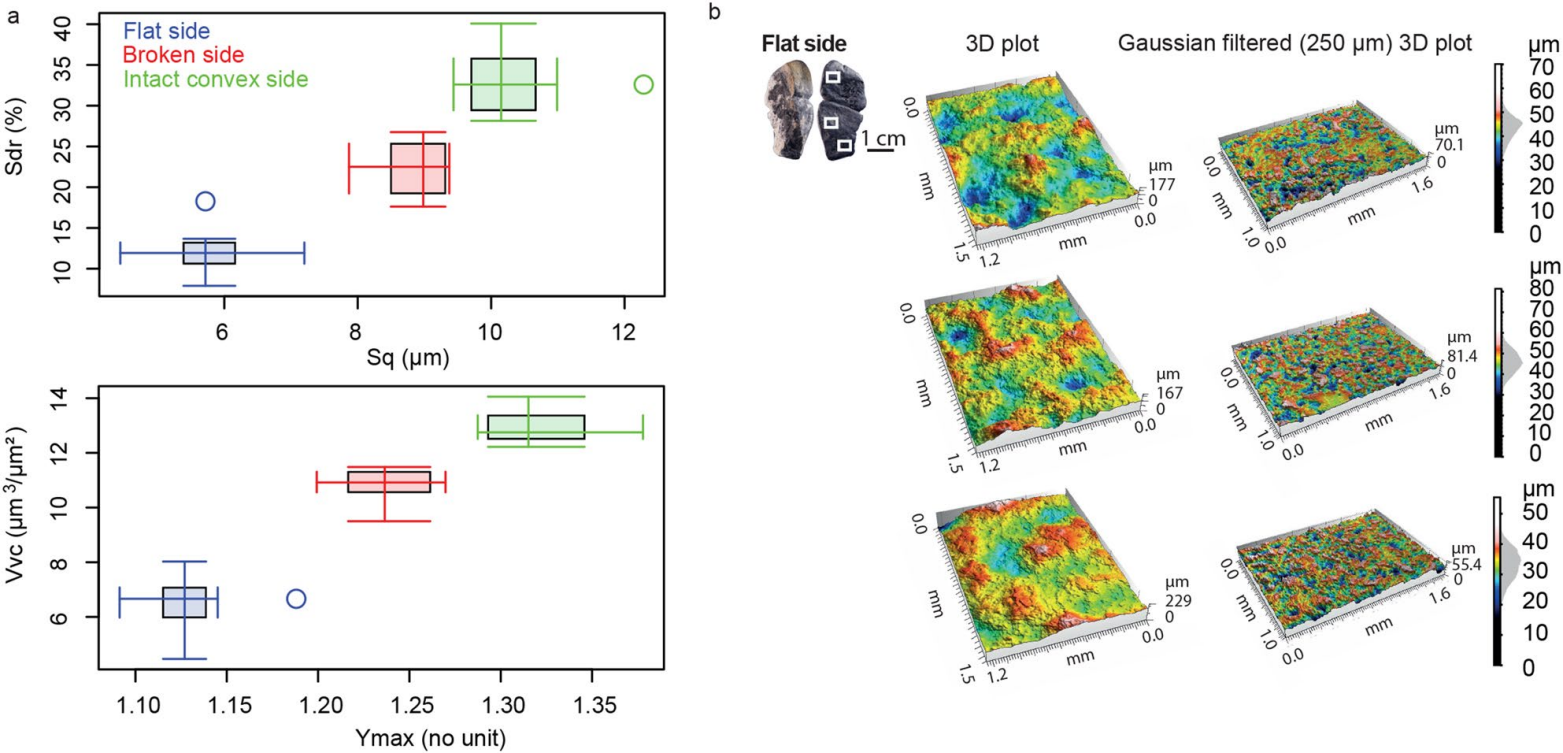
Surface roughness parameters registered on the graphite pendant with confocal microscopy: (**a**) Double boxplots showing the four more informative parameters registered with confocal microscopy on each surface of the pendant; (**b**) 3D views of the surface of flat side of the pendant produced by confocal microscopy. The 3D images in the right column are filtered with a Gaussian filter with a 250 µm cut-off. The positive skewness of the distribution is visible on the grey surface on the z scale representing the distribution of the height measurements.

Four variables are informative as to the roughness of the breakage, the intact convex side, and the flat side (Fig. 3a). The lowest roughness values, reflecting a smoother (Sq), less complex surface texture (Sdr, Asfc), were obtained on the flat side of the pendant (Fig. 3a and Supplementary information Fig. S4a). The 3D reconstructions of the surface show a relatively flattened surface with a restricted range of elevation values (Fig. 3b). It is consistent with the negative Ssk values indicating the presence of large plateaus and narrow valleys (Supplementary information Fig. S4a).

The convex side of the pendant yielded the highest roughness values, thereby reflecting an irregular complex surface texture (Fig. 3a and Supplementary information Fig. S4a). The 3D reconstructions of the surface show a relatively erratic surface, including large and deep voids (high Vvc values) and steep hills corresponding to a high range of elevation values (Supplementary information Fig. S4b). The broken part of the pendant shows medium values for all the parameters (Fig. 3a and Supplementary information Fig. S4a-c). The differences between the two sides primarily reflect different functional and taphonomic histories for the surfaces of the pendant.

The two grooves differ in profile, steepness, and ridge sharpness, thereby indicating that the mid-section groove has been used more intensely. The use-wear pattern and profile are consistent with suspension at the mid-section groove using a cord of either vegetal fibers, leather or sinew. The sharper groove at the extremity of the pendant, less affected by use-wear, forms a decoration engraved on the pendant with no discernable functional meaning.

The preservation of the flat side is good, with no calcite concretion and no breakages. When compared to the natural texture of the graphite observed on the broken part of the pendant, the roughness values are particularly low. Morphological and roughness attributes indicate the presence of an intense use-wear that resulted in a flattening of the surface and an apparent gloss. Overall, the use-wear pattern is consistent with suspension of the pendant in contact with soft material for an extended period. The convex side of the pendant, including the broken part, is less regular than the flat surface. The higher roughness values point at functional and taphonomic differences for a surface with an ancient breakage that was not in contact with the garment during the suspension.

Technological analysis indicates that the graphite pebble was flattened on one side by grinding, and one extremity was decorated by a short groove using a pointed stone tool. The pendant was suspended by a cord tied around a mid-section groove shaped using a similar tool. Rugosimetric analysis on the flat side is consistent with contact with a soft material, probably during suspension. Due to the absence of typical grinding facets and striations caused by repeated contacts between the artifact and an abrasive surface^51–53^, we exclude that the grinding process may correspond to extraction of graphite powder for use as a pigment. The broken part of the pendant does not show evidence of use but it is covered by calcite concretions. The concretion is also observed on the unbroken part of the convex side. The concretions suggest that the breakage is not recent, implying that the pendant was lost during its use after the breakage.

## Discussion

### Graphite sourcing

Having established the age and complex life-history of the object, the question of its origins is still open. Graphite sources are not documented in the direct vicinity of the site. A survey of the active streams surrounding the site (Tolbor and Selenga rivers) during the 2019 field season revealed no evidence for such material in secondary rock deposits along the banks, which are igneous rather than metamorphic. The Mineral Resources Authority of Mongolia has identified several large deposits and smaller occurrences of graphite sources, primarily located in the Eastern Sayan Mountains, south-southeast of Lake Khovsgol, and in the southern Khentii Mountains^54,55^. The large deposits in the southern Khentii and northern Gobi regions (Supplementary information Fig. S5, n° 3, 4, and 9–13); however, are at least 400–500 km away from the Tolbor Valley. Another potential source of graphite is located south of Lake Khovsgol, around 220–260 km from the Tolbor Valley, near the source of the Egiin Gol River, the western tributary of the Selenga River. Prehistoric human settlements, such as Dörölj 1, which include EUP assemblages, are found in the region at the river's mouth, about 80 km from the Tolbor River confluence^56^. Another graphite deposit is documented at Kholboo Uul (Supplementary information Fig. S5, n° 14), roughly 100 km away from the Tolbor archaeological sites. This deposit is in the Khanuy Gol Valley watershed, the eastern tributary of the Selenga River, where numerous Paleolithic sites have been identified^57^. Because, the Kholboo Uul graphite sources are relatively far from the Khanuy Gol fluvial basin (circa. 35 km), and with the Egiin Gol River flowing downstream to join the Selenga River, we consider fluvial transport of the raw material unlikely. Instead, we consider more parsimonious that the graphite used to create the pendants was transported, directly or not, from within the Selenga River Basin but at a distance exceeding hunter-gatherers’ daily foraging range. Overall, the stratigraphic position of the figurine within an EUP deposit that lacks worked or raw fragments of graphite, the exogenous origin of the material, and the presence of heavy use-wear on the pendant suggest that the latter was already in use when it was imported to the site.

### Pendant morphotype

The general shape, the groove, and the short incision at one extremity of the pendant are among the most salient features used to identify phallic representations in various regional and chronological contexts. A representation refers to the utilization of symbols and signs to substitute and act on behalf of something else^58^; in terms of artistic expression, "representation" includes the documentation of sensory data. How accurate the artistic representation is depends on its level of resolution and the stylistic conventions shared between the maker and the observers^59^.

Representations simplified at the limit of abstraction are common in the Prehistoric record. Yet, whether or not an object is on full display, the codification process of a symbol rests on known stylistic conventions understood by other group members. More generally, actual figures are often reduced to their most salient, recognizable attributes: Upper Paleolithic mammoth representations in caves depicted only by the double-curved back line of the animal (i.e. Rouffignac Cave, Font de Gaume, Pech-Merle, France^60^), Upper Paleolithic Venus depicted as highly schematic women’s bodies, only represented by vague curvy profiles (i.e. Monruz Switzerland, Gönnersdorf, Nebra, Germany^61^), or the stone bird from Lingjing represented by a simplistic small bird profile^10^.

Keeping in mind the small size of the object, the most salient features shared by the T21 pendant and other objects of this category are a short groove depicting the external urethral meatus and another groove for the neck (Supplementary information S8, Model3D Graphite pendant). These features—a groove at the mid-section and a short deep groove at one extremity—are observed on a limestone pebble found in the Early Aurignacian at Les Cottés (France)^62^ and a long pebble with a circular groove at Hohle Fels Cave (Germany), discovered in a layer dated to 28 ka ago. Although the long pebble was used as a retoucher, it is also interpreted as a phallus representation^63^. Long, cylindrical items with a groove for the neck and sometimes an incision for the meatus are unproblematically accepted as phallic representations in other archaeological contexts, for example the pebbles with a mid-section circular groove from the PPN sites of Atlit-Yam^64^, Kfar Hahoresh^65^, and Ahihud Junction^66^. Resembling the cylindrical short pestles^67^, they are interpreted as stone phalluses (Supplementary information Fig. S6). The sample of the 3D phallic representations available in the literature shows the same exaggeration of key anatomical traits that obviously evoke a phallus.

Although it is difficult entirely to rule out other possibilities, based on these morphological analogies the most compelling interpretation of the T21 pendant is a simplified phallic representation. Despite its older age, the T21 pendant fits within a symbolic repertoire shared with later EUP sites in a European context, for example Abri Castanet^68^ and Abri Blanchard^69^ (Dordogne, France), both of which produced phallus representations in layers attributed to the Aurignacian. Although comparable depictions are still unknown at such an early date in Eurasia, the T21 ornament materializes a widely used symbol, with a broad chronological and geographical distribution. During the later UP, phallic representations—both as isolated forms and as complete human male ithyphallic images^70^ depicted through cave art (paintings and engravings) and portable art^71,72^ — become more frequent in Europe^63,71^.

### Function of the pendant

Historically, the emergence of figurative and sexed representations has been considered to be a phenomenon restricted to western Europe, and associated with the dispersal of the earliest *Homo sapiens* populations and/or the development of new cognitive capacities^72–74^. Figurative art is not documented earlier than 37,000 years ago in Europe^33,68^ and 30,000 years ago in Africa^75^, although examples exist in Southeast Asia between *circa* 50,000–40,000 years ago^50,76^. The T21 phallic pendant is older than similar European examples by at least 5000 years, and of a similar age to estimates for figurative cave paintings from Borneo and Indonesia^76^.

The ornamental nature of the artifact from T21 and its male symbolism raises questions as to its social and ritual function. The exogenous origin of the material from which it was carved suggests movements and interactions with distant territories. The decoration on the pendant, which featured a short groove depicting the meatus, was probably difficult to notice when it was worn during everyday interactions. The Upper Paleolithic record, however, has provided many ornaments with subtle decorations. The Upper Paleolithic decorated red deer canines from Ningxia, China^77^, from Aven des Iboussières in France^78^, as well as small punctuations on Aurignacian ivory items and pendants^79,80^ are a few examples among many. The symbolic significance of these understated modifications, including those on the phallus-like pendant, was not necessarily linked to their visibility at a distance.

**Figure 4:**
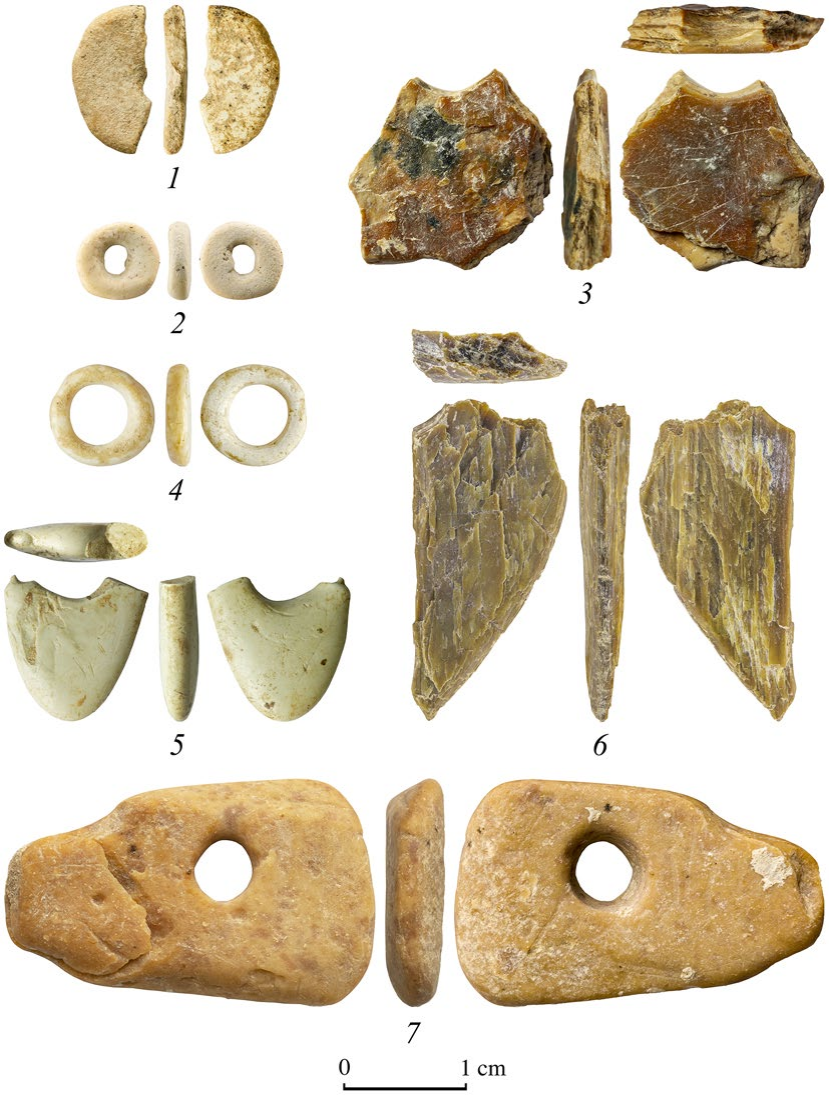
Personal ornaments and blanks from Upper Paleolithic assemblage AH4 at Tolbor-21 site: 1–4–ostrich eggshell beads ; 3, 6-serpentinite pendant and blank; 5-steatite bead; 7-pyrophyllite pendant.

In a European context, phallus representations have been interpreted in various ways, including as fertility or masculinity symbols^81^, apotropaic symbols^82^, evidence that societies already knew the biological principles of reproduction^63^, artifacts used for rites of passage related to the biological maturity of individuals, or even as objects of religious worship^83^. Although their social role is unclear, phallic symbols have been discussed in terms of group cohesion, individuation, and identity, or associated with general considerations around the emergence of social complexity, and the materialized aspects of symbolic thought, gender relations, and sexuality^70,84–88^. It is difficult to associate a specific function or meaning with the T21 pendant, but we note that current studies of human anthropomorphic sexed imagery—including the several hundred stone, bone, ivory, and antler anthropomorphic figurines known from a wide geographic and temporal range later in the UP^89–95^– seek to identify particular patterns of spatial and temporal variability and propose interpretations far beyond the dual matriarchy/patriarchy myth^63,68,91,96^ (Fig. 4).

### Regional comparisons

**Figure 5:**
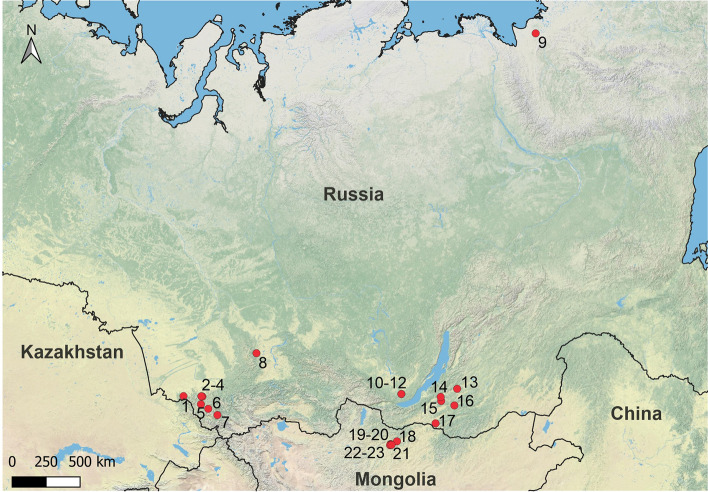
Regional comparisons. In red, location of the sites mentioned in Table 1 where personal ornaments attributed to the early stages of Upper Paleolithic have been documented. 1-Strashnaya cave, 2-Denisova Cave, 3-Anui 2; 4-Ust'‐Karakol 1, 5-Ust'‐Kan cave, 6-Kara‐Bom, 7-Maloyaloman cave, 8-Malaya Syia, 9-Yana RHS site, 10-Gerasimova I (Pereselencheskyi punkt), 11-Mamony II, 12-Shchapova I, 13-Khotyk, 14-Kamenka, 15-Varvarina gora, 16-Tolbaga, 17-Podzvonkaya, 18-Dorolj 1, 19-Tolbor-4, 20-Tolbor-21, 21-Kharganyn‐gol 5, 22-Tolbor-16, 23-Tolbor-17. Map was created with QGIS 3.8.1. Open Source Geospatial Foundation Project. http://qgis.osgeo.org.

Recent excavations and research on the UP from Gorny Altai and Transbaikal regions have yielded a rich and diverse record of symbolic objects (Table 1, Fig. 5). Evidence of personal ornament use is known as early as 45 ka cal BP in the Initial Upper Paleolithic at Kara Bom and Denisova Cave in Siberian Altai^97,98^ and Podzvonkaya in Transbaikal region^99^. The use of soft stones along with ostrich eggshell, ivory and bones has been documented at sites coeval with the AH4 assemblage from T21 (Fig. 4). It is attested in many EUP sequences from regions bordering Mongolia—Transbaikal and Cis-Baikal—as a tradition lasting until the end of the MIS3.

**Table 1:** Regional comparisons.

Region/Site	Unit	14C age *circa* cal. BP (68.3%)	Bone/antler/ivory	Tooth	OES	Other**	Soft stone	Cultural affiliation	References
*Transbaikal*									
Podzvonkaya	East Complex AH3	42.5–41.5	PP/1	–	P/12	–	PP^3^/2	Laminar IUP	^99^
	South East Complex AH5	Supposedly same as East Complex	–	–	P/5, Nd/1	–	–	Laminar IUP	^99^
	Lower Complex AH1-AH2	46–> 44	–	–	P/1	–	–	Laminar IUP/mixed	^99^
Khotyk	AH2	40–30.5	–	–	–	–	PP^2^/2	Laminar IUP?	^122^
	AH3	42–33.8	–	–	–	–	PP^2^/6, R^3^/2, B^3^/3	Laminar IUP	^122,123^
Kamenka	A(C)	44.4–34.9	B/3, Nd(?)/?	–	B/?	–	B^3^/3	Laminar IUP	^98,123,124^
Varvarina Gora	AH2	< 40.5–34	–	–	–	–	B^3^/1, PP^3^/1	Laminar IUP	^124^
Tolbaga	AH4	46–25.9	P/2	–	–	–	–	Laminar IUP/mixed with EUP	^125–128^
*Mongolia*									
Tolbor-4	AH5	< 44.3–35.8	–	–	B/2	–		Laminar IUP/mixed with EUP	^129,130^
Tolbor-16		38.5–35–8			B/4			Laminar EUP/UP	^105^, this study, Fig. S7
Tolbor-17		33–32			B/28	–	PP^3^/2	Laminar UP	this study, Fig. S7
Tolbor-21	AH4	42.9–41.4	–	–	P/1, B/2	–	PP^1^/2, PP^2^/1, P^5^/1, P^7^/1	Laminar IUP/EUP	^48^, this study
Kharganyn-gol-5	AH5	42.5	–	–	–	–	Nd^6^/1	Laminar IUP	^46^
Dorolj 1	dec-13	36.8–34.1	–	–	B/2	–	–	Laminar EUP	^56^
*Cis-Baikal*									
Gerasimova I (Pereselencheskyi punkt)	AH2	41.6–31.1	Nd^2^/1	–	–	–	PP^2^/2, R^2^/1, Nd^2^/1	Flake EUP	^131^
Mamony II	AH2	37.5–35.8	–	–	–	–	PP^2^/3	Flake EUP	^132^
Shchapova I	6	43.5	–	–	–	–	PP^3^/1	Flake EUP	^133^
*Central Siberia*									
Malaya Syia		34.5–30.1	–	–	–	–	UP^1^/7	MUP/EUP mixed	^134^
*Russian Altai*									
Denisova Cave	11.1 East Gallery	> 50–32.9	B/9, R/2, plaques/4; button/1; diadem/1	P/12	B/9	Nd/1	PP^1^/2, B^2^/1, B^3^/1, R^1^/1; BR^1^/1	MP/IUP/EUP	^7,40,98^
	11.2 East Gallery	> 50–41.3							
	11 Main Chamber	< 50–36							
Kara-Bom	UP2	46–45.5	B/2	B/1	–	–	–	Laminar IUP	^97^
Strashnaya cave		MIS3	–	P/?	–	–	–	Mixed	^135^
Maloyaloman cave	3	38	–	P/1	–	–	–	Laminar IUP	^97^
Ust'-Karakol 1	9	34.2–38	–	–	–	–	PP^1^/1 + Nd fragments	Laminar EUP	^45,47^
Anui 2		29.3–27	–	–	–	B/1	PP^2^/1	MUP/EUP	^40,98^
Ust'-Kan cave	?	mid-MIS3	P/1		–	–	–	MP/IUP/EUP mixed	^97^
*North Eastern Siberia*									
Yana RHS site		32.5–31.1	5891 complete beads and 1148 preforms	–	–	–	–	flake EUP	^136^

List of the personal ornaments found within Upper Paleolithic occupations in Mongolia and surrounding regions. P-pendant, PP-soft stone pendant with perforation, B-bead, R-ring, Nd-Not determined; BR-bracelet; 1-Serpentinite; 2-Talc; 3-Unknown; 4-sepiolite; 5-graphite; 6-muscovite; 7-pyrophyllite. The number of ornaments found follows the slash.

No detailed typological, technological and functional analysis is available yet for most of the personal ornaments identified within the IUP/EUP of the region. Soft stone pendants of various shape, size and color are widely documented in the region (Table 1), but OES beads have not been documented in the Cis-Baikal and North Eastern Siberia yet (Table 1). Mongolia is characterized by the lack of faunal remains compared to other regions, and animal teeth appear to be ornaments specific to the Russian Altai (Table 1). In this record, in contrast to the few ostrich eggshell beads and the soft stone pendants discovered in the same layer, the phallus-like pendant of T21 is a unique anthropomorphic representation for the regional Paleolithic record otherwise characterized by several bead types (ostrich eggshell beads, tubular bone beads) and stone pendants^45,100–105^ (Table1).

We can only speculate on the exact context and processes leading to this innovation, but unlike the European examples, the T21 pendant was produced in a period and region where our species encountered others. Chronologically, it overlaps with age estimates for the early introgression events between *Homo sapiens* and Denisovans^106–109^. Unlike the earliest *Homo sapiens* in Siberia (ca. 45 ka, Western Siberia)^44^, the Salkhit skull cap (34 ka, East Mongolia) and the Tianyuan Cave (40 ka, Northern China) do show evidence of Denisovan introgression^110^. Geographically, the Tolbor Valley is among the regions where physical encounters between the Denisovans and our species are plausible^49,106^ and where population dynamics may have constituted the breeding ground for cultural innovations.

### Conclusion

The T21 pendant, dating to *circa* 42 ka cal BP, provides new evidence of symbolic production and human self-representation previously unknown in the early phases of the Upper Paleolithic. The pendant and its cultural context are stratigraphically, chronologically and technologically intermediate between the IUP and the classic EUP. The pendant presented here suggests that three-dimensional images of the human body and symbolized sexed attributes were produced on portable objects during a period of early *Homo sapiens* dispersals in Eurasia.

## Materials and methods

### Raw material identification and functional analysis of the pendant

The raw material from which the pendant is manufactured was assessed by optical microscopy and micro-Raman spectrometry (µ-RS). We used a SENTERRA Dispersive Raman Spectrometer (Bruker), equipped with an internal calibration system. Spectra acquisitions were done via the OPUS 7.2 software, with a 532 nm laser, a laser power of 2mW, an integration time of 60 s, and multiple co-additions. Several locations of the artefact were tested, both on recent breakage and archeological surfaces. Decomposition of the spectra was also conducted in Opus 7.2. Other artefacts were characterized by petrographic examinations using a Zeiss AxioLab Pol polarizing microscope with transmitted light. Elemental composition was determined by means of a handheld Olympus Vanta M spectrometer. The location and extent of worked areas and the sequence of the technical actions were systematically recorded on the pendant based on microscopic examination.The artefact was examined at magnifications between 4 × and 40 × and photographed with a motorized Z6 APOA equipped with a DFC420 digital camera to document taphonomic and anthropic modifications. The functional analysis relies on experimental data replicating grinding, scraping and polishing stone raw material as well as use-wear by suspension and manipulation of various raw materials including stones^111–118^. Digital images were collected at different heights and adapted algorithms in the Multifocus module of the Leica Application Suite (LAS) were used in order to combine them into one single, sharp composite image that significantly extends the depth of field. Treatment of data by the Leica Map DCM 3D produces 3D reconstructions of areas of interest.

Selected areas of the pendant were scanned using a Sensofar S neox scanning confocal microscope using a 20 × objective allowing for a lateral resolution of 0,65 µm and a vertical resolution of 0,31 µm. Data was acquired in confocal fusion mode with SensoScan 6.7 and the resulting files were analyzed with SensoMap 7.4 software. Shape was removed by subtracting a third-degree polynomial, and isolated or around edges outliers were removed and non-measured points filled. A Gaussian filter was applied to these areas to separate roughness and waviness with a 250 µm cut-off value, and captured areas were subsequently divided into four 828 × 622 µm sub-areas. ISO 25,178 international standards were used to calculate different 3D area surface texture parameters for roughness^119,120^. Scale Sensitive Fractal Analysis (SSFA) was also performed on the unfiltered surface as a second way to obtain roughness parameters^121^. As a result of the application of both methods, 7 relevant roughness parameters were selected to document the state of the different surfaces of the artefact (Table S3). They characterize the distribution of the height of the filtered surface (Sq, Ssk, Sku), the complexity (Sdr), the volume of the voids (Vvc) and the fractal relative area and the fractal complexity (Ymax, Asfc).

3D-scanned of the pendant was performed using Einscan-SE and the associated software EXscan 3.1.0.1. The pendant was scan twice using a turntable, with 12 stops per scans. The automatic alignment was based on salient features and the mesh was built watertight and in high definition.

## Supplementary Information

Supplementary Information 1.

Supplementary Information 2.

## Data Availability

Data supporting the results can be found in the article.
