# Supplementary material for: Symbolic innovation at the onset of the Upper Paleolithic in Eurasia shown by the personal ornaments from Tolbor-21 (Mongolia)
Source: Sci Rep. 2023 Jun 12;13:9545. doi: 10.1038/s41598-023-36140-1 (PMC10261033; doi:10.1038/s41598-023-36140-1)
Supplement: Supplementary file 1 — Supplementary Information 1. [file 41598_2023_36140_MOESM1_ESM.pdf]

Click to activate the 3D content
in dedicated pdf viewer

3D model of the graphite pendant from Tolbor 21
Rigaud et al. 2023 Scientific Reports
