# Supplementary material for: Symbolic innovation at the onset of the Upper Paleolithic in Eurasia shown by the personal ornaments from Tolbor-21 (Mongolia)
Source: Sci Rep. 2023 Jun 12;13:9545. doi: 10.1038/s41598-023-36140-1 (PMC10261033; doi:10.1038/s41598-023-36140-1)
Supplement: Supplementary file 2 — Supplementary Information 2. [file 41598_2023_36140_MOESM2_ESM.pdf]

Supplementary Information for

Symbolic innovation at the onset of the Upper Paleolithic in Eurasia through the personal ornament fromTolbor-21 (Mongolia)

Solange Rigaud[1*], Evgeny P. Rybin[2*], Arina M. Khatsenovich[2], Alain Queffelec[1], Cleantha H. Paine[3], Byambaa Gunchinsuren[4], Sahra Talamo [5,6], Daria V. Marchenko[2], Tsedendorj Bolorbat[4], Davaakhuu Odsuren[4], Christopher J. Gillam[7], Masami Izuho[8], Alexander Yu. Fedorchenko[2], Dashdorjgochoo Odgerel[10], Roman Shelepaev[11], Jean-Jacques Hublin[12,5], Nicolas Zwyns [13,5]

[1]CNRS-Université de Bordeaux, UMR5199 PACEA Bâtiment B2 Allée Geoffroy Saint Hilaire, 33615 Pessac, France.

[2]Institute of Archaeology and Ethnography, Siberian Branch, Russian Academy of Sciences, 17 Lavrentiev ave., 630090, Novosibirsk, Russia.

[3]Archaeology Institute, University of the Highlands and Islands, Kirkwall, UK.

[4]Institute of Archaeology, Mongolian Academy of Sciences, Peace Avenue, Ulaanbaatar-13330, Mongolia

[5]Department of Human Evolution, Max Planck Institute for Evolutionary Anthropology, 04103, Leipzig, Germany.

[6]Department of Chemistry "G. Ciamician", University of Bologna, Via Selmi, 2, 40126, Bologna, Italy.

[7]Winthrop University, 701 Oakland Ave, Rock Hill, SC 29733, USA.

[8]Faculty of Social Sciences and Humanities, Tokyo Metropolitan University, Hachioji, Tokyo 192-0397, Japan.

[10]Institute of Geology, Mongolian Academy of Sciences, Ulaanbaatar, 15160, Mongolia.

[11]V.S. Sobolev's Institute of Geology and Mineralogy, Siberian Branch of Russian Academy of Science. Russia, Novosibirsk 630090, Ak. Koptyug avenue 3.

[12]Chaire de Paléoanthropologie, Collège de France, 75005 Paris, France.

[13]Department of Anthropology, University of California-Davis, 1 Shields Avenue, Davis, CA 95616.

*Solange Rigaud
Email: solange.rigaud@cnrs.fr ; solange.rigaud@u-bordeaux.fr

*Evgeny P. Rybin
Email: rybep@yandex.ru

Supplementary Information Text

S1. Initial and Early Upper Paleolithic in Eurasia

Originally, the concept of Initial Upper Paleolithic (IUP) was introduced to describe the first Upper Paleolithic emerging out of a transitional process at Boker Tachtit, in the Negev desert [1]. The definition was later broadened to include assemblages showing a combination of Levallois and volumetric blade production beyond the Levant [2], that can be (but does not have to be) transitional[3]. In Central and East Asia, the IUP is recognized starting from 48-45 ka cal BP and is defined based on a recurring combination of techno-typological derived traits relative to the local Middle Paleolithic. IUP lithic assemblages consistently show a production of large blades, often relying on a specific method known as asymmetrical core reduction. Some of the large blades are segmented and further reduced as burin-cores, and these two methods are integrated in a coherent technical system[4]. In North Mongolia, the best examples of such technology are documented at Tolbor-4 and Tolbor-16. The IUP is, however, well documented between the Altai, the Transbaikal and North Mongolia, with possible examples further south and east. At the scale of Eurasia, the IUP is considered as evidence for the dispersal of *Homo sapiens* and the subsequent encounters with Neanderthal populations[5]. Assemblages sometimes include clear examples of personal ornament [6–8].

A more diverse Early Upper Paleolithic appears in the same regions around 40 ka cal BP. Just like in other parts of Eurasia, it is characterized by a shift toward smaller laminar formats produced by percussion and often referred to as ‘bladelets’[9]. Although core reduction methods may vary, assemblages have in common the production of small blanks absent in the IUP. This replaces the burin-core method and comes as a complement to generic blades, following specific reduction pathways (e.g. Tolbor-15 OH-6 and OH7, Tolbor-21, AH3)[10]. When found in stratified contexts, the EUP is always above the IUP (e.g. Tolbor-4, Tolbor-16), and because of its chronological position it is attributed to *Homo sapiens* populations. In the valley, it also includes ornaments such as OES beads or stone pendants[11,12]. AH4 at T21 is exceptional regionally for being chronologically, stratigraphically and typo-technologically intermediate between the IUP and the EUP[13]. At a broader scale, the age of 42 ka cal BP overlaps with the IUP in Eastern Europe (e.g.

Bacho-Kiro)[14]; hence with *Homo sapiens* populations having recent Neanderthal ancestry. These seem to lack descent in present-day European populations, but descendants are identified in the present-day populations of Asia[15]. It suggests that IUP populations survived in Asia, and AH4 could be illustrating such continuity, or alternatively, be archaeological evidence of another population movement. Either ways, it also overlaps chronologically with the development of the EUP in the Levant[16,17] and pending further description of the lithic material, we will refer to AH4 here as EUP.

S2. Sedimentology and stratigraphy.

Landscape position

T21 comprises 2 test pits and 4 larger pits excavated in a southward-sloping landform (Figure 1 S1 a and b). Pit 2, where the pendant was discovered, is located on the eastern edge of the landform close to a Holocene gully cut into unconsolidated upper Pleistocene deposits which mantle a bedrock hill. A <4 m-deep test trench (Pit 3) revealed a deep sequence of low- to moderate-energy slope deposits capped by a thin deposit of eolian silt and the Holocene soil complex. The slope deposits are generally fine-grained, and their accumulation is the result of climatically driven cyclic alternation between the deposition of laminar silts (eolian deposition, pedogenesis, colluviation in the broad sense, and episodic carbonate surface crust formation, with occasional higher-energy episodes), and the downslope displacement of packs of these sediments by solifluction. The individual lithostratigraphic units remain in chronological order, but truncation and the occasional action on a given sediment of multiple episodes of solifluction might rarely cause the mixing of sediments of different ages.

Description of lithostratigraphic units

The 2016 excavations at Pit 2 exposed approximately 170 cm of soil, eolian silt, and soliflucted laminar silt with occasional gravel and cobbles (Figure S1e). The matrix material throughout the section consists of fine and very fine silt. Coarse sand is angular and looks very fresh; most of it is of local origin, and some is derived from the mechanical weathering, during frost action and solifluction, of the larger clasts in the section, which

originate in a nearby bedrock outcrop and arrive through gravitational input. The sediments can be subdivided into three main units: the Holocene soil (unit 1; 20-30 cm), eolian silt and reworked or soliflucted eolian silt (unit 2; 40-60 cm), and a laminar soliflucted silt with sand, gravel, and cobbles (unit 3), which is the top of the long sequence of laminar soliflucted deposits described above; finer division into lithostratigraphic units (L.U.) is as follows:

1.1: A thin medium-brown loose silt loam with sand and gravel, with abundant fine roots. The A horizon of the Holocene soil, a Kastanozem (FAO classification) (10 cm). Abundant burrows extend to a depth of 50cm.

1.2 A firm, pale precipitate over 1.3 and the upper part of 2.1. An incipient Bk horizon associated with the Kastanozem, with secondary carbonate accumulation as micritic enrichment of the matrix (5–10 cm).

1.3: A faint grey-brown silt with gravel below the A horizon, mostly visible as fine root marks extending a few cm into the underlying L.U.; these may represent an earlier soil (truncated) (5 cm).

2.1: Pale yellow dense, firm eolian silt affected in its upper half by calcium carbonate accumulation related to the overlying Holocene soil complex (30 cm).

2.2: Soliflucted soft pale brown loess-like sediments with gravels (20–30 cm). The solifluction episode(s) affecting this LU also involve(s), to a limited extent, the sediments of the underlying L.U. 3.1.

3.1: Firm white (carbonate-rich) and pale brown flat-lying laminar silt with a platy structure. Laminae throughout unit 3 are compositional (varying carbonate and organic matter content) or textural. A line of flat-lying gravels and occasional cobbles at the unit top dips parallel to the present-day slope (10 cm). L.U. 3.1 appears to truncate the underlying L.U. 3.2 and 3.3.

3.2: Loose, grey massive silt present only in a few patches between 3.1 and 3.3 (10–20 cm).

3.3: Loose grey-brown silt with coarse sand (10 cm), affected by solifluction and with occasional faint laminarity (8-10cm).

3.4: Soliflucted pale (carbonate-rich) and medium brown firm finely laminar silt with sparse inclusions of coarse sand and gravel (15–30 cm).

3.5: A thin pack of pale brown (carbonate-rich) laminar sandy silt with abundant sand and gravel involved in solifluction together with the overlying 3.4, but whose presence is variable (10 cm).

3.6: Medium brown massive sandy silt with gravel, present in patches (10 cm).

3.7 Intensely cryoturbated loose, light and medium brown silt with frequent coarse sand and gravel, sometimes forming tails around decomposing cobbles of intensively mechanically weathered local bedrock. Original laminae distorted by cryoturbation. Elongate clasts are frequently vertical (>30 cm).

AH4 and the position of the artifact

The age of the phallus pendant is constrained only by its stratigraphic context, and so this section explores the sedimentary processes recorded in Pit 2 with a view to understanding how these might have affected its position. Of these processes (eolian deposition, weak soil development, colluviation, and solifluction), solifluction is the most significant in terms of artifact displacement, which was evidently mostly lateral (downslope). At T21, the mixing of sediments of different ages is only possible where multiple L.U.s are affected collectively by a single episode of solifluction. This is the case with units 3.2 and 3.3, whose solifluction appears also, toward the northern end of the section, to involve the surface of the underlying L.U. 3.4; in this case older artifacts from the top of L.U. 3.4 may lie within younger sediments. This is also theoretically possible with L.U. 3.4-3.6, collectively involved in an episode of solifluction, but in fact no mixing is observed. Because the accumulation of fairly thick packs of sediment alternates with episodes of solifluction, the opposite scenario, where younger material lies within older sediments, is not recorded within Pit 2 and is difficult to envisage for the site as a whole. Bioturbation (as burrows) at Pit 2 is limited to L.U.1 and the upper part of L.U. 2, well above the position of the artifact.

The phallus pendant was discovered near the southeast corner of Pit 2 and is associated with archaeological horizon (AH) 4, an accumulation of lithics and a few fragments of bone distributed throughout the laminar silt matrix of L.U. 3.4 (Figure S1c and d). The accumulation of L.U. 3.4 was dominated by low-energy processes primarily affecting particles less than 2 mm in size and unlikely to have affected larger clasts, but three

overlapping solifluction lobes incorporating this and some of the underlying L.U.s are visible in the section (Figure S1f). The sediments in the southeast corner of the site probably represent the upslope area (back) of a solifluction lobe, where vertical displacement would have been minimal; the riser (frontal ridge) is probably downslope, beyond the edge of the pit. The laminarity of the sediment is intact and shows sediment movement to have been mostly lateral. The stratigraphic sequence in Pit 2 is broadly intact without stacking, repetition, mixing or large-scale sediment or artifact displacements; this is especially true for the southern part of the section, where the artifact was found. There can be no question of downward movement of artifacts from overlying units anywhere within L.U. 3.4; the episode of solifluction affecting AH4 takes place before the deposition of AH3, so that any sediment mixing could only involve older, not younger, sediments. Given that low-energy slope processes predominate in the formation of the laminar silt, and given the position of the phallus pendant in an area which experienced only slight movement during solifluction (away from the front of the lobe), the artifact is likely to be in situ within AH4, and the age range for AH4 gives a minimum age for the artifact (upward movement from the underlying AH5 is theoretically possible, but there is no evidence for this).

Considered within their sedimentary context, the radiocarbon dates for Pit 2 are in stratigraphic order and increase with depth. The deepest dated sample, MAMS 31817, dates the top of L.U. 3.4, which houses AH4; the sediment has flowed over itself during solifluction in this part of the pit (see Fig. S1f), and this sample would originally have lain at around the same level as the now-overlying MAMS 31816, which yields a similar date. The underlying LU 3.6 is dated by MAMS 31817, which yields the oldest date.

S3. Lithic assemblage of Pit 2, AH4

The archaeological assemblage from AH4 (Pit 2) includes 1468 artifacts, and 470 lithics categorized as debris (assemblage from Pit 2 AH 4 and excavated in 2016 – 2017 is described in Rybin et al., 2020). Fine- and very fine grained siliceous rocks, , were the principal raw material employed with primary outcrops located within a 300 m radius of Pit 2. With 43% of laminar blanks (Fig. 1b n°1, 4-7, 10) AH4 is a blade-based assemblage, and it includes the debitage associated with such production. The primary core reduction

strategy is documented by subprismatic and flat cores, both frequently reduced following an asymmetrical method [13,18]. Located next to the raw material outcrop, the assemblage documents primary core preparation and reduction steps. The largest blades were produced from two opposed, alternating, striking platforms. The volumetric reduction strategy, which is adaptive to the shape of raw material nodules, reflects the use of convex core working surfaces, frequently shifting from a wide flaking surface to the core's narrow face and then back again (asymmetrical reduction method). Cores for flake production and several blanks indicate the presence of Levallois technology. There is a high frequency of retouched tools (15%), half of which (55%) were made on laminar blanks.

The tool types include sidescrapers (Fig. 1b n°8), end-scrapers on blades (Fig. 1b n°4, 7), rare bifaces (Fig. 1b n°9), various retouched blades and points (Fig. 1b n°5, 6, 10), including points with trimmed transverse edges and truncated points. Chronologically, the assemblage post-dates the Central Asian and South Siberian Initial Upper Paleolithic. The latter appears and spread throughout southern Siberia and Central Asia around 50,000 – 45,000 radiocarbon years ago. AH4 differs technologically from the "classic" IUP assemblages, such as Kara Bom in Russian Altai, Kamenka A in Transbaikalia and Tolbor 4 and 16, situated in the Tolbor Valley [18–21]. IUP reduction strategy, such as the burin-cores for small blade production, is absent while typical asymmetric core reduction is less prominent than in IUP assemblages. Specific, elongated, bidirectional pointed blades are rare.

The assemblage stands out as a phase previously unreported in the Valley and beyond, technologically and chronologically intermediate between the underlying Initial Upper Paleolithic (IUP) and the Early Upper Paleolithic horizons[13,18]. Pending further analysis, AH4 is described here as Early Upper Paleolithic (EUP) in the broad sense. Assemblage of overlying AH 3 represents a typical North Mongolian EUP. Laminar forms still occupy 45.8% in blank production, but with a different morphology: large pointed bidirectional blades disappeared, while the number of bladelets has increased – which, along with specialized bladelet cores, reflect a general trend toward miniaturization. Unidirectional core reduction technology predominates, while in reduction technology; asymmetric cores are lacking

S4. Radiocarbon

Pretreatment and dating

The samples were pretreated at MPI-EVA Leipzig using a previously published method described in Talamo and Richards (2011)[22,23]. Approximately 500 mg of the whole sample were first cleaned and then demineralized in 0.5 M HCl at room temperature until no CO_2 effervescence could be observed. 0.1 M NaOH was then added for 30 min to remove humics. The NaOH step was followed by further rinsing with 0.5 M HCl for 15 min. The sample was then gelatinized, following Longin 1971[24], in a pH3 solution at 75 ℃ for 20 h. The resulting gelatin was first filtered in an Eeze-Filter™ (Elkay Laboratory Products (UK) Ltd.) to remove small (<8 µm) particles and then through a 30 kDa ultrafilter (Sartorius "Vivaspin 15")[25]. Prior to use, the filter was cleaned to remove carbon-containing humectants[23]. The sample was then lyophilized for 48 h. Samples were selected for dating when showing a satisfactory amount of collagen yield (>1%) [23,26] and C:N between 2.9 and 3.5 [23,27]. The selected samples were dated by AMS at the Klaus-Tschira-Labor für Physikalische Altersbestimmung (Curt-Engelhorn- Zentrums für Archaometrie), Mannheim, Germany [28].

Bayesian Model

The Bayesian model has been made based on the radiocarbon dates from Rybin *et al*. 2020. All information regarding the dated bone samples are available in the publication[13]. Based on the radiocarbon dates obtained in Pit 2 at Tolbor-21 (for other pits, also see [13]), the Bayesian Model was built using the new IntCal20 [29] in the Oxcal 4.4 program [30]. Results are shown in Tables S1, S2, and Figure S2. For each model, a General t-type Outlier Model was computed to detect problematic samples with prior probabilities set at 5%. The 'date' command was used to establish the probability distribution functions (PDFs) ranges, e.g., start and end boundaries, as well as to the duration of the AH 4 layer.

S5. Raman spectroscopy

Raman spectra (Figure S3) show a weak D1 band, very weak D1+G bands and strong G band and S1 bands. Compared to reference data [31,32], the Raman analysis identifies the raw

material as a well-crystallized graphite. The spectra obtained from the Tolbor-21 pendant are almost identical to the reference data published in Sole et al. (2014, fig. 3) with the band S1 named 2D and decomposed in 2D(1) and 2D(2). This raw material is far different from charcoal, lignite, or other carbon black material commonly found in other archaeological contexts [34–37], but very similar to the material excavated from Scladina Cave [38]. The exact same graphite spectrum was obtained for each location analyzed on the black surface of the artifact. Based on the areas of the decomposed bands and on the equation from Beyssac et al. [39], it is possible to calculate an approximated value of ca. 550-560 °C as the maximum temperature undergone by carbon material during metamorphism (Table S3). This value allows us to associate this carbonaceous material with high-grade metamorphism of amphibolite or hornblende hornfels facies.

The white concretion covering part of the surface is identified by µ-RS as calcite, with characteristic bands at 1086, 712 and 281 cm^{-1}.

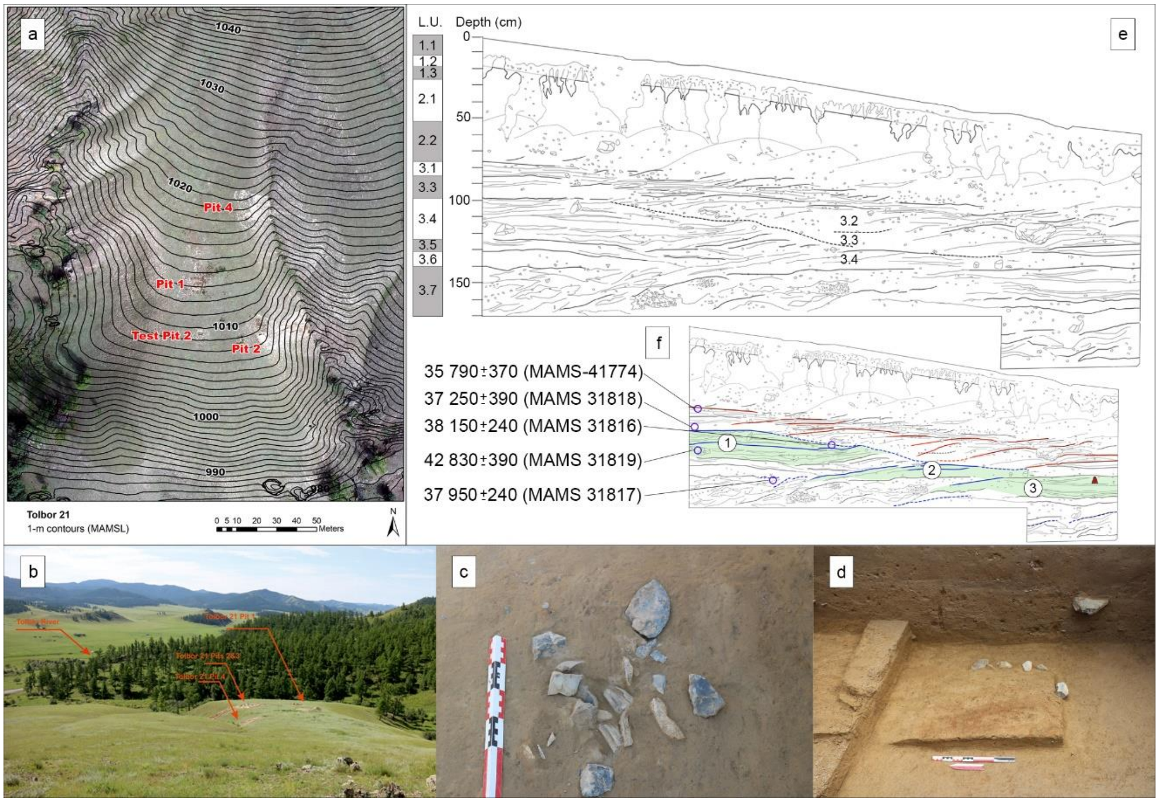

Fig. S1. Landscape, sedimentology, and stratigraphy at T21.

a, Contour map showing the position of Pits 1-4 at T21. **b**, View from above the site showing the landform and the location of the pits at T21. **c,** A detail of AH4, the archaeological context of the phallus pendant, showing lithics and bone fragments lying within fine-grained deposits. **d,** A detail of AH4 showing a lobate arrangement of lithic material surrounding a patch of darker sediments within L.U. 3.4. Sediment analysis is currently underway to identify any evidence of burning within these darker patches. **e,** Stratigraphic drawing of the east wall of Pit 2, where the pendant was discovered. The sediments are divided into three main units, with individual lithostratigraphic subdivisions indicated on the left margin and within the drawing. Within unit 3, the laminar sediment, bold lines indicate observed divisions between lithostratigraphic units, while finer lines indicate laminarity. Dashed lines indicate stratigraphic divisions inferred from interruptions in the laminarity of the deposits. **f,** Post-depositional sediment movements within unit 3 at Pit 2; numbered circles indicate individual solifluction lobes incorporating L.U. 3.4 (green shading) which houses AH4. Blue lines: upper boundaries of solifluction lobes incorporating L.U. 3.4-3.6. Areas of less certainty, for example the lower boundaries of the lobes, are indicated by dashed lines. Red lines: upper boundaries of solifluction lobes incorporating the sediments of 3.2 and 3.3; dashed lines indicate areas of less certainty. The solifluction event affecting L.U. 3.4 (blue lines) took place before the deposition of the overlying L.U. 3.2 and 3.3 (red lines). The position of the pendant is indicated by a red triangle, and the positions of dated samples are indicated by blue circles (2m projection).

Fig. S2. Bayesian Model for Tolbor-21, Pit 2. The model has been produced based on the dates listed in Rybin et al. (2020). Radiocarbon dates were calibrated and modelled using IntCal20 (Reimer et al. 2020) in the Oxcal 4.4 program (Ramsey 2009). Outliers' prior and posterior probabilities are shown in square brackets.

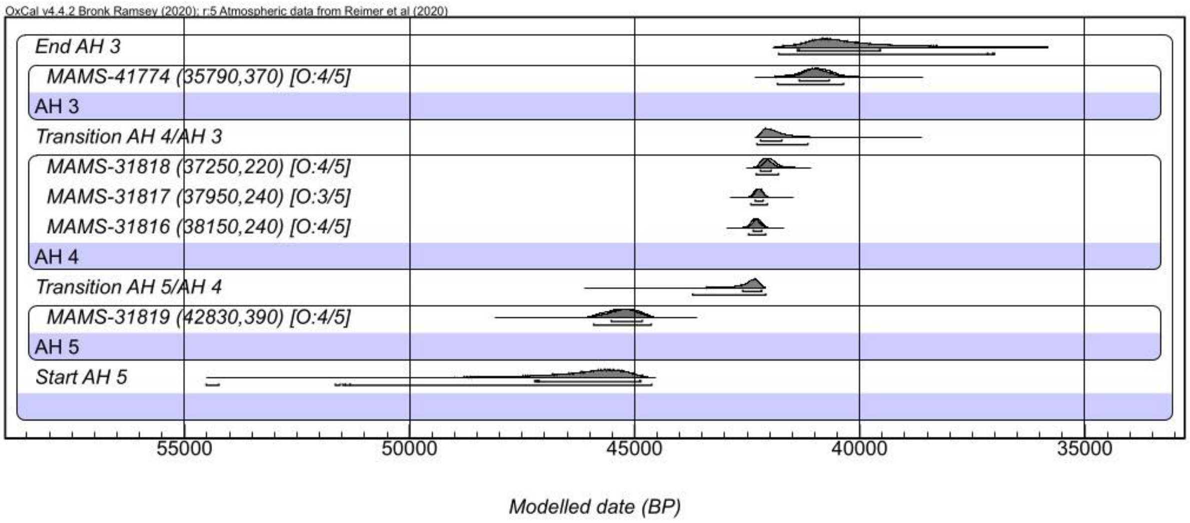

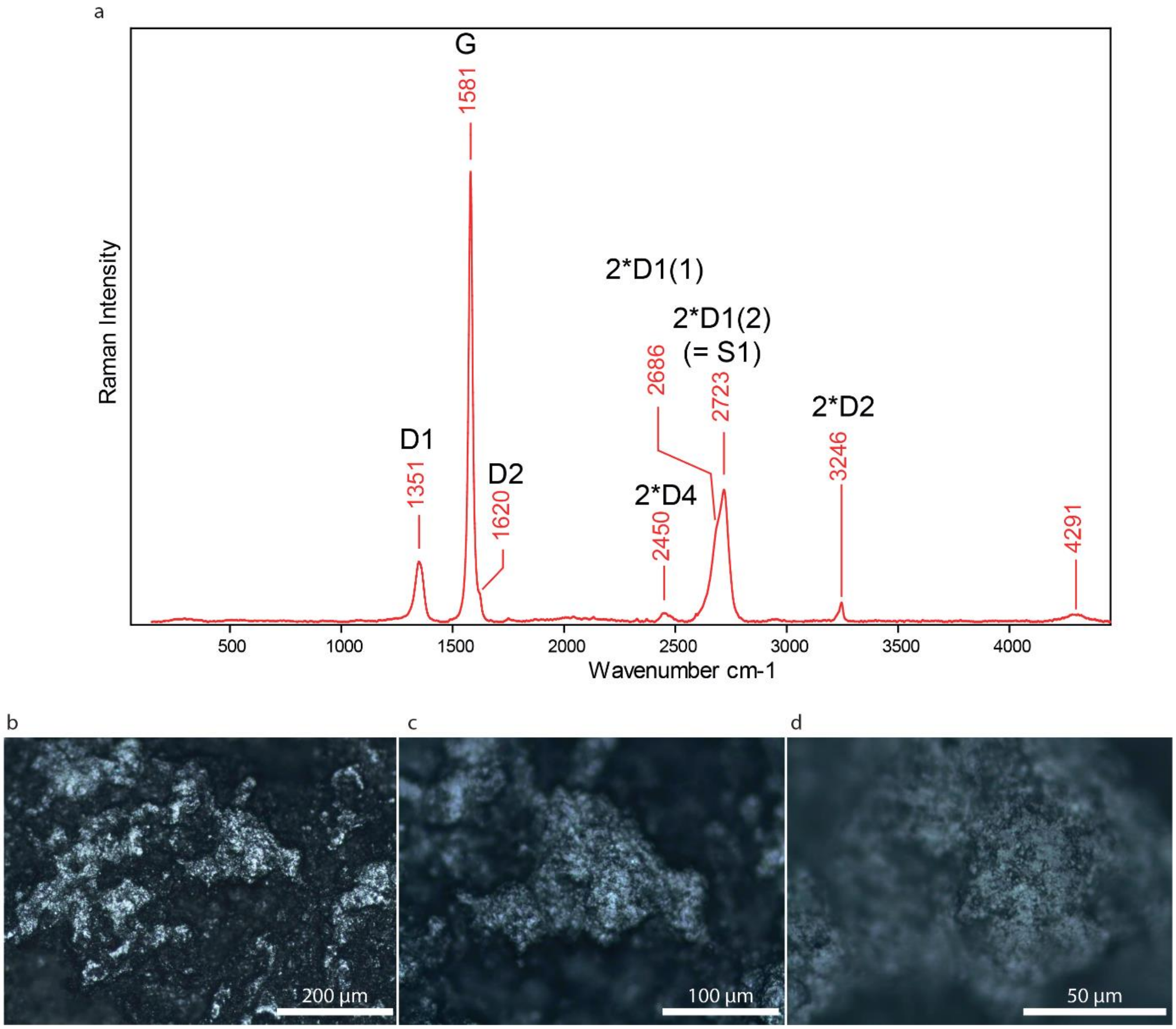

Fig. S3. Raman spectroscopy.

a, The Raman spectra obtained on the Tolbor artifact identifies the raw material as being well crystallized graphite. The spectra shows very strong G band, strong 2*D1 (also called S1) bands, and a weak D1 band. G stands for Graphite, while D stands for disordered carbon **b**, **c**, **d**, microphotographs of the surface of the Tolbor artifact submitted to the Raman acquisitions, made respectively with the 10x, 20x and 50x objectives of the microRaman spectrometer.

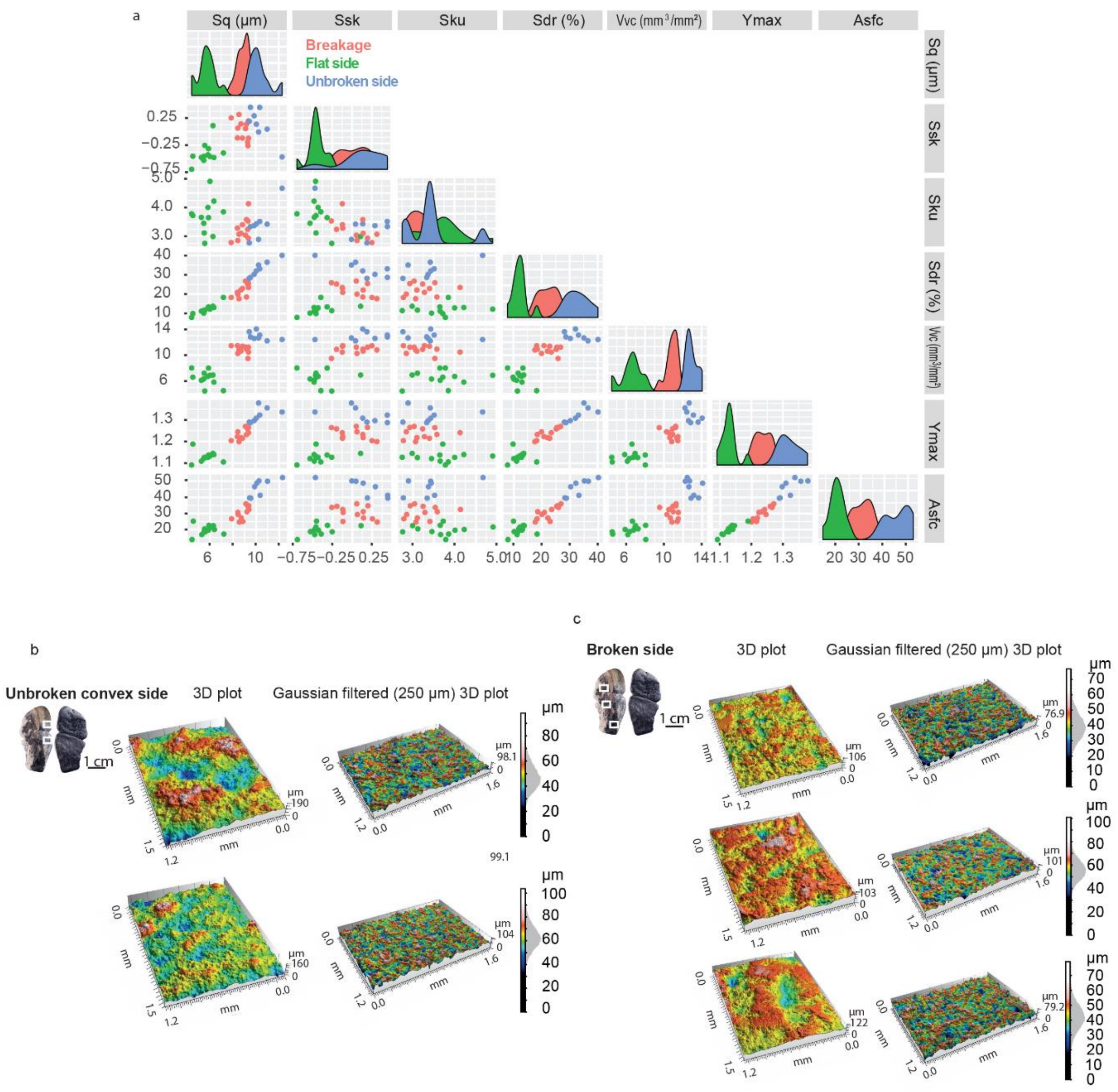

Fig. S4. Tribological study of the surface of the artifact.

a, Biplots showing the distribution of the roughness parameters registered at different locations on the flat, convex side and broken surface of the pendant. Distribution frequency of each parameter is showcased in the diagonal; **b, c**, 3D views of the convex (b) and broken (c) sides of the artifact measured by confocal microscopy. A Gaussian filter with a 250µm cut-off has been applied to the 3D images in the right column. The positive skewness of the height measurement distribution is represented by the frequency curve shaded in grey along the z scale. The filtered surface of the convex side and the breakage show a heterogeneous texture with high to moderate hills and voids.

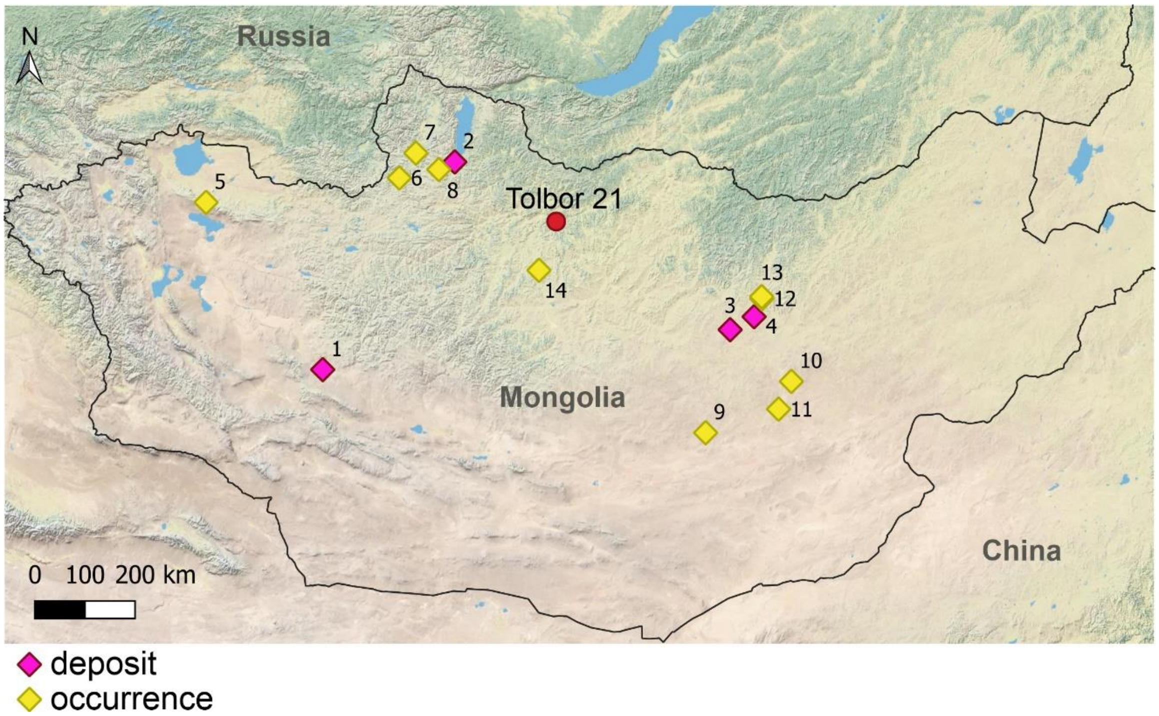

Fig. S5. Distribution map of graphite sources in Mongolia. Deposits: 1 - Urd khujiriin gol, 2 - Khargana gol, 3 – Zulegt, 4 – Jargalant. Occurrences: 5 – Khayrgas, 6 - Khokh khujirt, 7 – Balbrik, 8 - Ovor maraat, 9 – Unnamed, 10 – Naidvar, 11 - Khukh tav, 12 - Baits uul, 13 - Shine bulagiin khondii, 14 – Kholbo uul. Map was created with QGIS 3.8.1. Open Source Geospatial Foundation Project. http://qgis.osgeo.org

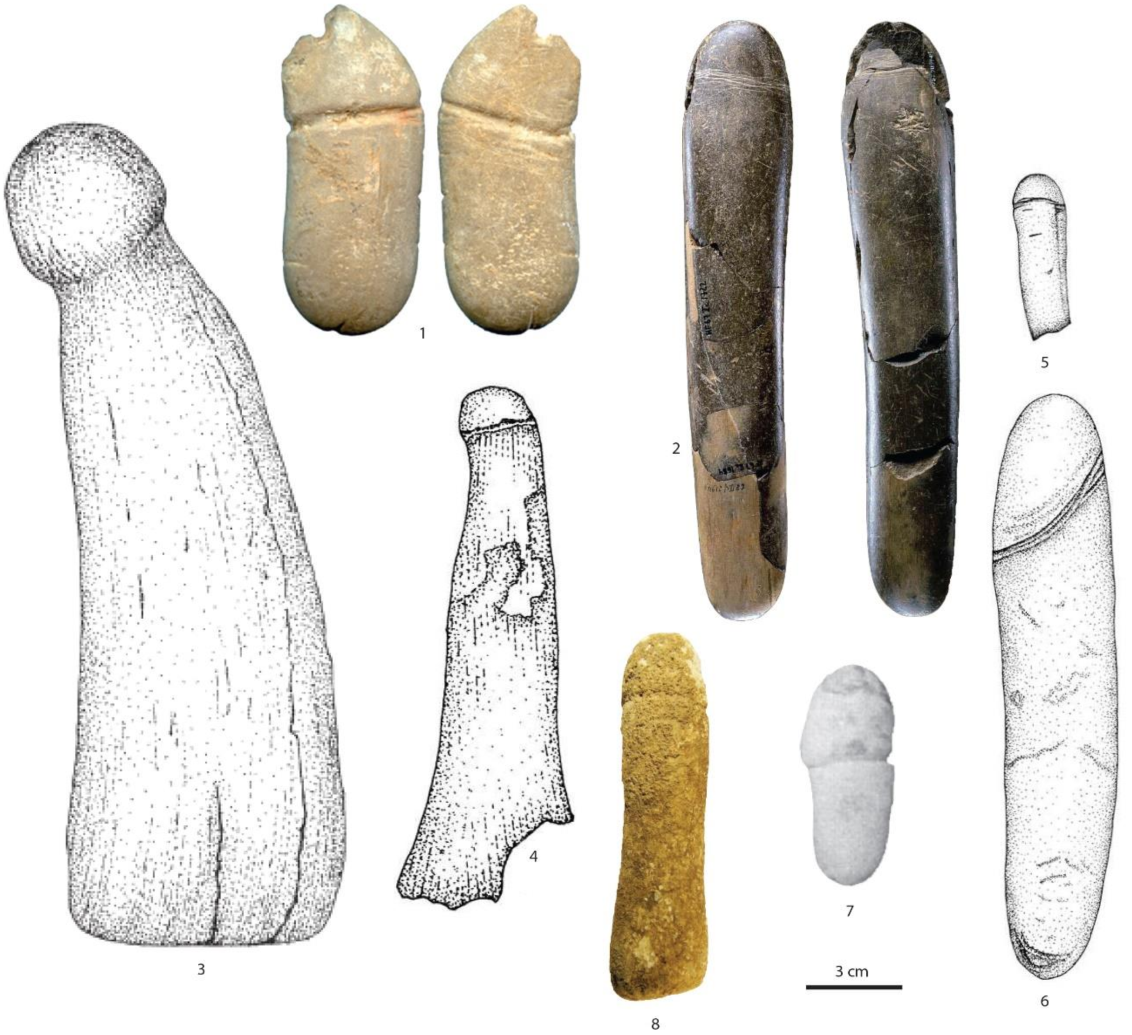

Fig. S6. Phallus 3D representations documented in the literature (modified after Feustel 1971, Galili *et al.* 1993, White 1993, Cook 2003 , Delluc and Delluc 2004, Conard and Kieselbach 2006, Goring-Morris *et al*. 2008, Rigaud *et al*. 2014,): 1) Les Cottés, France, Early Aurignacian, limestone[40], 2) Hohle Fels Cave, Germany, Gravettian, stone[41], 3) Abri Blanchard, France, Aurignacian, bison horn core[42], 4) La Combe, France, Aurignacian[43], 5) Abri Pataud, France, Gravettian, limestone[44], 6) Oelknitz, Germany, Magdalenian, stone[45], 7) Atlit-Yam, Carmel Coast, PPNB, limestone[46], 8) Kfar HaHoresh, Lower Galilee, PPNB, limestone[47]. The pendant discovered at les Cottés was not described as a phallus in the publication, but the artefact show similar grooves than the Tolbor pendant.

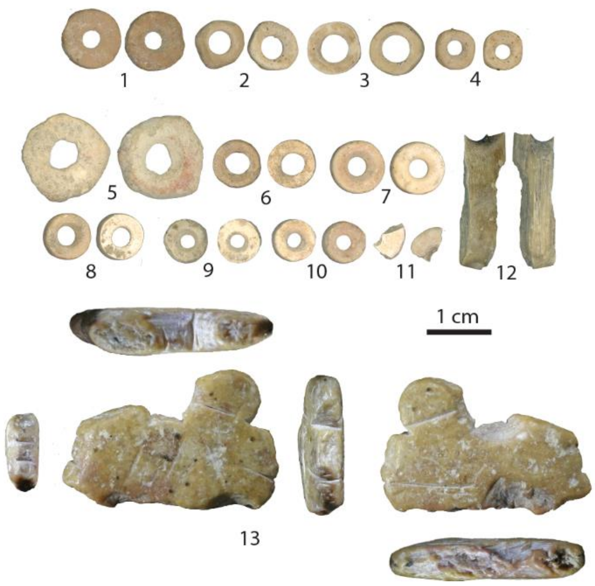

Fig. S7. Personal ornaments from Tolbor 16 (1-4) and Tolbor 17 (5-13). 1-11 : OES beads ; 12 : fragment of serpentinite pendant ; 13: calcite pendant.

Table S1. Radiocarbon dates and % of Collagen, Tolbor-21, Pit 2. Only bones have been dated and, in the Valley, their preservation is such that cortical bones is often in bad shape. The bones were found in direct association with the archaeological material, and our strategy was to date geological units (and when possible, solifluction lobes) to provide an age for the material in the layer. The C:N values are measured using the Thermo Scientific Flash Elemental Analyzer, coupled to a Delta V isotope ratio mass spectrometer.

**Submitter Code**	**Block excavation**	**Archaeological Horizon**	**Collagen yield (%)**	**C/N ratio**	**Lab number**	**^{14}C Age**	**Error (1σ)**
7174-3A	Pit 2	3	8.8	3.2	MAMS-41774	35,790	370
T21-5878	Pit 2	4	12.9	3.1	MAMS-31816	38,150	240
T21-6345	Pit 2	4	7.7	3.2	MAMS-31817	37,950	240
T21-5452	Pit 2	4	11.7	3.2	MAMS-31818	37,250	220
T21-6301	Pit 2	5	11.9	3.1	MAMS-31819	42,830	390

Table S2. Calibrated dates and Bayesian Modelled results of all the 14C ages obtained from Pit 2 at Tolbor-21. In red is the duration calculated by the Date Command in OxCal.

**Tolbor 21**	**Un-modelled (BP)**				**Modelled (BP)**			
Indices<br>Amodel 95.7<br>Aoverall 96.1	**from**	**to**	**from**	**to**	**from**	**to**	**from**	**to**
	**68,30%**		**95,40%**		**68,30%**		**95,40%**	
**End AH 3**					**41390**	**39530**	**41810**	**37000**
MAMS-41774 (35790;370)	41240	40590	41580	40080	41350	40670	41830	40350
**Transition AH 4/AH 3**					**42210**	**41720**	**42290**	**41150**
MAMS-31818 (37250;220)	42130	41860	42240	41640	42210	41960	42300	41800
MAMS-31817 (37950;240)	42360	42150	42450	42040	42330	42140	42420	42050
MAMS-31816 (38150;240)	42420	42220	42520	42110	42370	42170	42470	42080
**Transition AH 5/AH 4**					**42600**	**42170**	**43720**	**42080**
MAMS-31819 (42830;390)	45650	44940	45980	44690	45530	44830	45910	44620
**Start AH 5**					**47230**	**44860**	**54520**	**44620**
**Tolbor 21**	**Modelled (Cal BP)**							
Indices<br>Amodel 95.7<br>Aoverall 96.1	**from**	**to**	**from**	**to**				
	**68,30%**		**95,40%**					
Transition AH 4/AH 3	42210	41720	42290	41150				
Duration of AH 4	42410	41950	43020	41380				
Transition AH 5/AH 4	42600	42170	43720	42080				

Table S3. Tribological analysis. Surface roughness parameters used to characterize the different surfaces of the graphite pendant with confocal microscopy

Parameter	Source	Unit	Description
Sq	ISO 25178	µm	Root Mean Square Roughness. Standard deviation of the height distribution that quantifies the statistical distribution of height values around the mean plane.
Ssk	ISO 25178	no unit	Skewness of the distribution of the Z values. Third statistical moment, qualifying the symmetry of the height distribution. A highly negative value indicates for example that the surface is mainly composed of plateaus with deep and fine valleys.
Sku	ISO 25178	no unit	Kurtosis of the distribution of the Z values. Fourth statistical moment, qualifying the flatness of the height distribution. High values represent spiked distribution, indicating avery sharp range of height distribution.
Sdr	ISO 25178	%	Developed interfacial area ratio. A high value indicates a complex surface that, once completely flattened, is far wider than the measured field of view.
Vvc	ISO 25178	mm^3/mm^2	Core void volume, as represented on the Abbott-Firestone curve, with p= 10% and q = 80% (Blateyron, 2013)
Ymax	SSFA	no unit	Maximum relative area. Calculated as the maximum area of all the triangles fitting the measured surface for each specific surface of the individual triangles.
Asfc	SSFA	No unit	Area-scale Fractal Complexity. Indicates the speed at which the relative area increases while downsizing the triangles used to fit the surface. It is a proxy of the complexity of the surface.

SI References

1. Marks, A. E. & Ferring, P. Changing core reduction strategies: A technological shift from the Middle to the Upper Paleolithic in the southern Levant. in *The Mousterian legacy: Human biocultural change in the Upper Pleistocene* (ed. Trinkaus, Erik.) 13–34 (1988).
2. Kuhn, S. L., Stiner, M. C. & Güleç, E. Initial Upper Palaeolithic in south-central Turkey and its regional context: a preliminary report. *Antiquity* **73**, 505–517 (1999).
3. Kuhn, S. In what sense is the Levantine Initial Upper Paleolithic a 'transitional' industry? *Trabalhos de Arqueologia* **33**, 61–69 (2003).
4. Zwyns, N. The Initial Upper Paleolithic in Central and East Asia: Blade Technology, Cultural Transmission, and Implications for Human Dispersals. *Journal of Paleolithic Archaeology* **4**, 19 (2021).
5. Hublin, J.-J. *et al.* Initial Upper Palaeolithic Homo sapiens from Bacho Kiro Cave, Bulgaria. *Nature* **581**, 299–302 (2020).
6. Kuhn, S. L., Stiner, M. C. & Reese, D. S. Ornaments of the earliest Upper Palaeolithic : News insights from the Levant. *Proceedings of the National Academy of Science* **98**, 7641–7646 (2001).
7. Zwyns, N. & Lbova, L. V. The Initial Upper Paleolithic of Kamenka site, Zabaikal region (Siberia): A closer look at the blade technology. *Archaeological Research in Asia* **17**, 24–49 (2019).
8. Martisius, N. L. *et al.* Initial Upper Paleolithic bone technology and personal ornaments at Bacho Kiro Cave (Bulgaria). *Journal of Human Evolution* **167**, 103198 (2022).
9. Hublin, J.-J. The modern human colonization of western Eurasia: when and where? *Quaternary Science Reviews* **118**, 194–210 (2015).
10. Zwyns, N. Laminar technology and the onset of the Upper Paleolithic in the Altai, Siberia. (Leiden University, 2012).
11. Zwyns, N. *et al.* The open-air site of Tolbor 16 (Northern Mongolia): Preliminary results and perspectives. *Quaternary International* **347**, 53–65 (2014).

12. Khatsenovich, A. M. *et al.* Long distance transport and use of mica in the Initial Upper Paleolithic of Central Asia: An example from the Kharganyn Gol 5 site (northern Mongolia). *Journal of Archaeological Science: Reports* **31**, 102307 (2020).
13. Rybin, E. P. *et al.* A new Upper Paleolithic occupation at the site of Tolbor-21 (Mongolia): Site formation, human behavior and implications for the regional sequence. *Quaternary International* (2020) doi:10.1016/j.quaint.2020.06.022.
14. Fewlass, H. *et al.* A 14C chronology for the Middle to Upper Palaeolithic transition at Bacho Kiro Cave, Bulgaria. *Nature Ecology & Evolution* **4**, 794–801 (2020).
15. Hajdinjak, M. *et al.* Initial Upper Palaeolithic humans in Europe had recent Neanderthal ancestry. *Nature* **592**, 253–257 (2021).
16. Alex, B. *et al.* Radiocarbon chronology of Manot Cave, Israel and Upper Paleolithic dispersals. *Science Advances* **3**, e1701450 (2017).
17. Kadowaki, S. *et al.* Lithic Technology and Chronology of Initial Upper Paleolithic Assemblages at Tor Fawaz, Southern Jordan. *Journal of Paleolithic Archaeology* **5**, 1 (2021).
18. Zwyns, N. *et al.* The Northern Route for Human dispersal in Central and Northeast Asia: New evidence from the site of Tolbor-16, Mongolia. *Scientific Reports* **9**, 11759 (2019).
19. Shunkov, M. V., Kozlikin, M. B. & Derevianko, A. P. Dynamics of the Altai Paleolithic industries in the archaeological record of Denisova Cave. *Quaternary International* **559**, 34–46 (2020).
20. Derevianko, A. P. & Shunkov, M. V. Formation of the Upper Paleolithic traditions in the Altai. *Archaeology, Ethnology and Anthropology of Eurasia* **3**, 12–40 (2004).
21. Derevianko, A. P. *et al.* Archaeological research of multilayer site Tolbor-4 in 2006. in *The Materials of Annual Session of Institute of Archeology and Ethnography SB RAS* 114 (2006).
22. Talamo, S. & Richards, M. A Comparison of Bone Pretreatment Methods for AMS Dating of Samples >30,000 BP. *Radiocarbon* **53**, 443–449 (2011).
23. Talamo, S., Fewlass, H., Maria, R. & Jaouen, K. “Here we go again”: the inspection of collagen extraction protocols for 14C dating and palaeodietary analysis. *STAR: Science & Technology of Archaeological Research* **7**, 62–77 (2021).

24. Longin, R. New Method of Collagen Extraction for Radiocarbon Dating. *Nature* **230**, 241–242 (1971).
25. Brown, T. A., Nelson, D. E., Vogel, J. S. & Southon, J. R. Improved Collagen Extraction by Modified Longin Method. *Radiocarbon* **30**, 171–177 (1988).
26. Ambrose, S. H. Preparation and characterization of bone and tooth collagen for isotopic analysis. *Journal of Archaeological Science* **17**, 431–451 (1990).
27. van Klinken, G. J. Bone Collagen Quality Indicators for Palaeodietary and Radiocarbon Measurements. *Journal of Archaeological Science* **26**, 687–695 (1999).
28. Kromer, B., Lindauer, S., Synal, H.-A. & Wacker, L. MAMS – A new AMS facility at the Curt-Engelhorn-Centre for Achaeometry, Mannheim, Germany. *Nuclear Instruments and Methods in Physics Research Section B: Beam Interactions with Materials and Atoms* **294**, 11–13 (2013).
29. Reimer, P. J. *et al.* The IntCal20 Northern Hemisphere Radiocarbon Age Calibration Curve (0–55 cal kBP). *Radiocarbon* **62**, 725–757 (2020).
30. Bronk Ramsey, C. Dealing with Outliers and Offsets in Radiocarbon Dating. *Radiocarbon* **51**, 1023–1045 (2009).
31. Beyssac, O. *et al.* On the characterization of disordered and heterogeneous carbonaceous materials by Raman spectroscopy. *Spectrochimica Acta Part A: Molecular and Biomolecular Spectroscopy* **59**, 2267–2276 (2003).
32. Cuesta, A., Dhamelincourt, P., Laureyns, J., Martínez-Alonso, A. & Tascón, J. M. D. Raman microprobe studies on carbon materials. *Carbon* **32**, 1523–1532 (1994).
33. Sole, C., E. Drewett, N. & J. Hardwick, L. In situ Raman study of lithium-ion intercalation into microcrystalline graphite. *Faraday Discussions* **172**, 223–237 (2014).
34. Coccato, A., Jehlicka, J., Moens, L. & Vandenabeele, P. Raman spectroscopy for the investigation of carbon-based black pigments. *J. Raman Spectrosc.* **46**, 1003–1015 (2015).
35. Jaubert, J. *et al.* Early Neanderthal constructions deep in Bruniquel Cave in southwestern France. *Nature* **534**, 111 (2016).

36. Naudinot, N. *et al.* Divergence in the evolution of Paleolithic symbolic and technological systems: The shining bull and engraved tablets of Rocher de l'Impératrice. *PLOS ONE* **12**, e0173037 (2017).

37. Tomasini, E. P., Halac, E. B., Reinoso, M., Di Liscia, E. J. & Maier, M. S. Micro-Raman spectroscopy of carbon-based black pigments. *Journal of Raman Spectroscopy* **43**, 1671–1675 (2012).

38. Bonjean, D. *et al.* A new Cambrian black pigment used during the late Middle Palaeolithic discovered at Scladina Cave (Andenne, Belgium). *Journal of Archaeological Science* **55**, 253–265 (2015).

39. Beyssac, O., Goffé, B., Chopin, C. & Rouzaud, J. N. Raman spectra of carbonaceous material in metasediments: a new geothermometer. *Journal of Metamorphic Geology* **20**, 859–871 (2002).

40. Rigaud, S. *et al.* Les pratiques ornementales à l'Aurignacien ancien dans le Centre-Ouest de la France : l'apport des fouilles récentes aux Cottés (Vienne). *Bulletin de la Société Préhistorique française* **111**, 21–40 (2014).

41. Conard, N. J. & Kieselbach, P. Ein phallusförmiges Steinwerkzeug aus den Gravettienschichten des Hohle Fels : ein Beitrag zur Deutung paläolithischer Sexualdarstellungen. *Archäologisches Korrespondenzblatt* **36**, 455–472 (2006).

42. Cook, J. Vom Mutterleib zum Sexualpartner. Sexuelle Bildsprache in der Kunst der Altsteinzeit. In: V. van Vilsteren / R.-M. Weiss (Hrsg.), 100 000 Jahre Sex. Liebe und Erotik in der Geschichte (Stuttgart 2003/2004) 6-13. in *100 000 Jahre Sex. Liebe und Erotik in der Geschichte* (eds. van Vilsteren, V. & Weiss, R. M.) 6–13 (Theiss, 2003).

43. White, R. *Préhistoire*. (1993).

44. Delluc, B. & Delluc, G. L'art à l'Abri Pataud (Les Eyzies, Dordogne). in *L'art du Paléolithique supérieur. Actes du colloque 8.2 et 8.3, Congrès de l'UISPP, Liège 2001* (eds. Lejeune, M. & Welté, A.-C.) vol. 107 87–94 (2004).

45. Feustel, R. Sexuologische Reflexionen über jungpaläolithische Objekte. *Alt-Thüringen* **11**, 7–46 (1971).

46. Galili, E. *et al.* Atlit-Yam: A Prehistoric Site on the Sea Floor off the Israeli Coast. *Journal of Field Archaeology* **20**, 133–157 (1993).

47. Goring-Morris, A. N. *et al.* The 2007-8 Excavation Seasons at Pre-Pottery Neolithic B Kfar HaHoresh, Israel. *Antiquity* 318 (2008).
